# Setting research priorities for global pandemic preparedness: An international consensus and comparison with ChatGPT’s output

**DOI:** 10.7189/jogh.14.04054

**Published:** 2024-02-16

**Authors:** Peige Song, Davies Adeloye, Yubraj Acharya, Danladi Adamu Bojude, Sajjad Ali, Rowalt Alibudbud, Sheri Bastien, Francisco Becerra-Posada, Monika Berecki, Adams Bodomo, Florencia Borrescio-Higa, Marie Buchtova, Harry Campbell, Kit Yee Chan, Sohaila Cheema, Mickey Chopra, Darien Alfa Cipta, Lina Diaz Castro, Kurubaran Ganasegeran, Teshome Gebre, Anton Glasnović, Christopher J Graham, Chinonso Igwesi-Chidobe, Per Ole Iversen, Bismeen Jadoon, Giuseppe Lanza, Calum Macdonald, Chulwoo Park, Mohammad Mainul Islam, Suleiman Mshelia, Harish Nair, Zhi Xiang Ng, Mila Nu Nu Htay, Kabiru Olusegun Akinyemi, Michelle Parisi, Smruti Patel, Prince Peprah, Ozren Polasek, Renata Riha, Elena S Rotarou, Emma Sacks, Konstantin Sharov, Srdjan Stankov, Wenang Supriyatiningsih, Rosnah Sutan, Mark Tomlinson, Alexander C Tsai, Dialechti Tsimpida, Sandro Vento, Josipa Vlasac Glasnović, Laura B Vokey, Liang Wang, Kerri Wazny, Jingyi Xu, Sachiyo Yoshida, Yanfeng Zhang, Jin Cao, Yajie Zhu, Aziz Sheikh, Igor Rudan, International Society of Global Health (ISoGH)

**Affiliations:** 1 School of Public Health and Women’s Hospital, Zhejiang University School of Medicine, China; 2 School of Health & Life Sciences, Teesside University, UK; 3 Department of Health Policy and Administration, The Pennsylvania State University, USA; 4 Gombe State University, Gombe, Nigeria; 5 Department of Medicine, Ziauddin Medical University, Karachi, Pakistan; 6 Department of Sociology and Behavioral Sciences, De La Salle University, Manila, Philippines; 7 Norwegian University of Life Sciences, Ås, Norway; 8 Public Health Development Organization, El Paso, USA; 9 School of Medicine, University of Zagreb, Croatia; 10 African Studies, University of Vienna, Austria; 11 Universidad Adolfo Ibañez, Santiago, Chile; 12 Olomouc University Social Health Institute, Palacký University, Olomouc, Czechia; 13 Centre for Global Health, Usher Institute, University of Edinburgh, UK; 14 School of Social Sciences, Monash University, Australia; 15 Weill Cornell Medicine – Qatar, Doha, Qatar; 16 The World Bank, Washington, USA; 17 Universitas Pelita Harapan, Jakarta, Indonesia; 18 National Institute of Psychiatry Ramón de la Fuente Muñiz, Mexico City, Mexico; 19 Seberang Jaya Hospital, Ministry of Health, Malaysia; 20 The Task force for Global Health, Addis Ababa, Ethiopia; 21 Croatian Institute for Brain Research, Zagreb University School of Medicine, Zagreb, Croatia; 22 Faculty of Biology, Medicine and Health, The University of Manchester, Manchester, UK; 23 University of Bradford, UK; 24 University of Nigeria, Enugu Campus, Nigeria; 25 Department of Nutrition, University of Oslo, Norway; 26 Egyptian Representative, Committee of Fellows of Obstetrics and Gynaecology, Oxford, UK, and Royal Berkshire Hospital, NHS, UK; 27 Oasi Research Institute-IRCCS, Troina, Italy; 28 University of Catania, Italy; 29 Department of Public Health and Recreation, San José State University, San Jose, California, USA; 30 University of Dhaka, Bangladesh; 31 Jos University Teaching Hospital, Nigeria; 32 School of Biosciences, Faculty of Science and Engineering, University of Nottingham Malaysia, Semenyih, Malaysia; 33 Department of Community Medicine, Faculty of Medicine, Manipal University College Malaysia, Melaka, Malaysia; 34 Lagos State University, Ojo, Lagos, Nigeria; 35 Clemson University, USA; 36 Editor, Journal of Global Health Reports, Washington, USA; 37 Social Policy Research Centre/Centre for Primary Health Care and Equity, University of New South Wales, Sydney, Australia; 38 Croatian Science Foundation, Zagreb, Croatia; 39 Algebra University College, Zagreb, Croatia; 40 Royal Infirmary of Edinburgh, University of Edinburgh, UK; 41 Universidad San Sebastián, Santiago, Chile; 42 Johns Hopkins Bloomberg School of Public Health, Baltimore, USA; 43 Koltzov Institute of Developmental Biology of Russian Academy of Sciences, Moscow, Russia; 44 Pasteur Institute, Novi Sad, Novi Sad, Serbia; 45 Children and Mother Health Movement Action, Yogyakarta, Indonesia; 46 Universiti Kebangsaan Malaysia Medical Centre, Kuala Lumpur, Malaysia; 47 Stellenbosch University, Cape Town, South Africa; 48 Massachusetts General Hospital, Boston, USA; 49 Department of Public Health, Policy and Systems, The University of Liverpool, UK; 50 University of Puthisastra, Phnom Penh, Cambodia; 51 Department of Hematology, Dubrava University Hospital, Zagreb, Croatia; 52 Guangdong Provincial People’s Hospital, Guangzhou, China; 53 Children's Investment Fund Foundation, London, UK; 54 School of Health Humanities, Peking University, Beijing, China; 55 World Health Organization, Geneva, Switzerland; 56 Capital Institute of Pediatrics, Beijing, China; 57 School of Information Science and Technology, Hangzhou Normal University, Hangzhou, China; 58 Usher Institute, University of Edinburgh, UK

## Abstract

**Background:**

In this priority-setting exercise, we sought to identify leading research priorities needed for strengthening future pandemic preparedness and response across countries.

**Methods:**

The International Society of Global Health (ISoGH) used the Child Health and Nutrition Research Initiative (CHNRI) method to identify research priorities for future pandemic preparedness. Eighty experts in global health, translational and clinical research identified 163 research ideas, of which 42 experts then scored based on five pre-defined criteria. We calculated intermediate criterion-specific scores and overall research priority scores from the mean of individual scores for each research idea. We used a bootstrap (n = 1000) to compute the 95% confidence intervals.

**Results:**

Key priorities included strengthening health systems, rapid vaccine and treatment production, improving international cooperation, and enhancing surveillance efficiency. Other priorities included learning from the coronavirus disease 2019 (COVID-19) pandemic, managing supply chains, identifying planning gaps, and promoting equitable interventions. We compared this CHNRI-based outcome with the 14 research priorities generated and ranked by ChatGPT, encountering both striking similarities and clear differences.

**Conclusions:**

Priority setting processes based on human crowdsourcing – such as the CHNRI method – and the output provided by ChatGPT are both valuable, as they complement and strengthen each other. The priorities identified by ChatGPT were more grounded in theory, while those identified by CHNRI were guided by recent practical experiences. Addressing these priorities, along with improvements in health planning, equitable community-based interventions, and the capacity of primary health care, is vital for better pandemic preparedness and response in many settings.

The emergence of coronavirus disease 2019 (COVID-19) precipitated an unparalleled global crisis that exposed the fragility of health systems and poor pandemic planning worldwide [1,2]. The pandemic led to job losses, disrupted education, exacerbated food insecurity, and strained health care systems, even in nations once lauded as the champions for pandemic and emerging infections readiness [3-5]. Notably, the pandemic highlighted and exacerbated disparities in health care access and outcomes between high-income countries (HICs) and low- and middle-income countries (LMICs) [6–8]. According to the latest World Health Organization (WHO) statistics, as of 5 July 2023, LMICs accounted for over 58% of COVID-19 deaths globally [9]. Although the WHO has declared that COVID-19 is no longer a global health emergency, the pandemic's impact will be long-lasting, which underscores the need for research to inform pandemic preparedness globally.

The COVID-19 pandemic highlighted the pivotal role of preparedness and governance in managing infectious disease outbreaks [10]. Numerous studies have demonstrated that these factors are independently associated with the severity of the pandemic. While some HICs delivered an effective response, LMICs have endured a disproportionate burden. In particular, the pandemic revealed the fragility of health systems in many nations and their lack of readiness to tackle infectious disease outbreaks. This underscores a pressing need for establishing resilient and coordinated systems for detecting, preventing, and responding to such crises. Furthermore, the pandemic has intensified existing health disparities globally, placing marginalised communities and vulnerable populations at heightened risk of infection, severe illness, and death [10,11]. Indeed, in many settings, poor, voiceless and marginalised population groups were the most affected, underpinning that addressing key social determinants, such as poverty and inequity, is equally important to tackle future pandemics and strengthen global health security [12,13].

Defining research priorities for global pandemic preparedness is crucial, particularly for LMICs, which contend with persistent underinvestment and limited resources in global health. Such prioritisation can inspire leaders, researchers, and stakeholders to tackle this burden, guide policymakers, and inform funding organisations with evidence-based interventions for enhancing pandemic preparedness in these settings. The process of setting research priorities has played a vital role in shaping the global health agenda in the 21st century. Previous approaches to priority-setting have included surveys [14,15], expert input [16], the Delphi method [17], and mixed methods [18]. The Child Health and Nutrition Research Initiative (CHNRI) method has emerged as the most widely used, transparent, systematic, and replicable prioritisation process that can also engage all relevant stakeholders [19–21]. It has successfully facilitated decision-making and consensus development in diverse fields of global health, making it a valuable tool for determining research priorities in post-COVID-19 global pandemic preparedness [21-26].

In this study, we used the CHNRI method to establish global research and development priorities for pandemic preparedness. We also used output from the Chat Generative Pre-trained Transformer (ChatGPT), version 3.5 (OpenAI, SF, USA) to compare human-produced and artificial intelligence (AI)-generated research priorities. ChatGPT presented us with a remarkable opportunity to compare the input crowdsourced from the group of experts to that of AI. Previous research based on the CHNRI method occasionally raised questions on whether the results by some other group of experts would provide a different set of priorities. This is the first study to compare the results of human collective opinion (assembled through the CHNRI process) and human collective knowledge (as captured in and generated by ChatGPT).

## METHODS

### Overview of the CHNRI method

**Figure 1:**
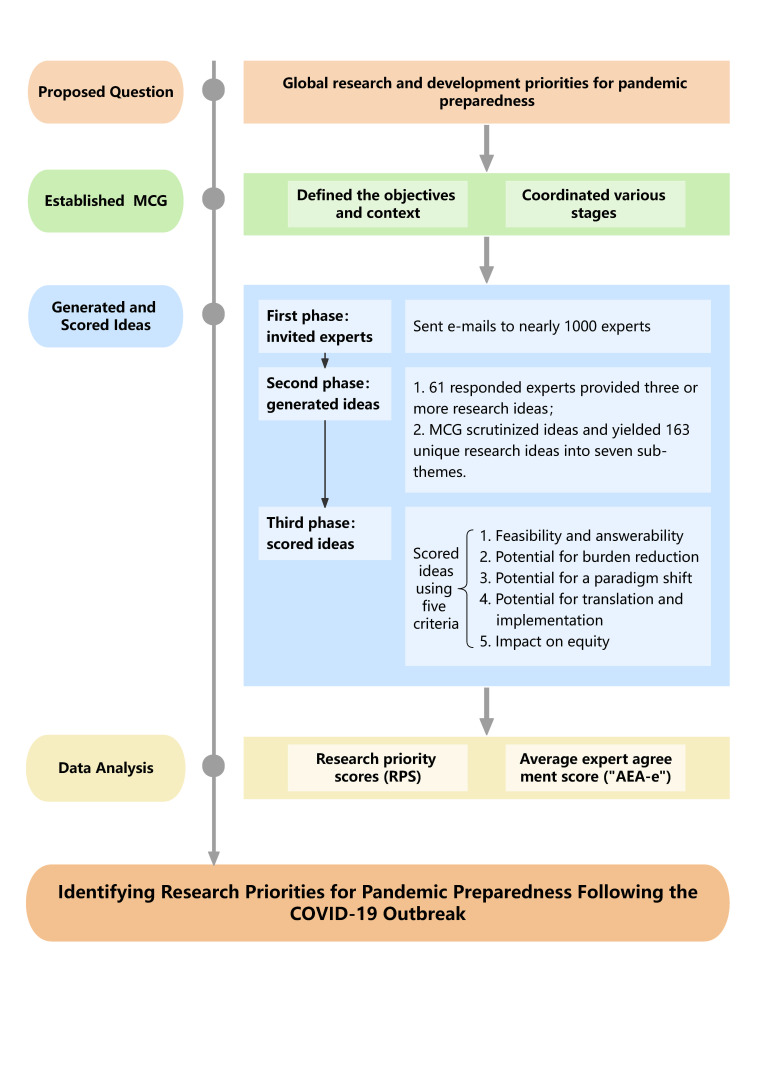
The process of CHNRI exercise.

In this study, we adapted the CHNRI method for prioritising research for pandemic preparedness. The CHNRI method is a systematic, transparent, and democratic approach that has been implemented in over 130 exercises led by national governments, funders, research institutions, and multilateral organisations such as the WHO and United Nations Children’s Fund (UNICEF) [21]. It uses crowdsourcing to generate independent research ideas from a group of experts, which are then scored against a predefined set of criteria [27]. Its advantages lie in its systematic nature; transparency and replicability; clearly defined context and criteria; involvement of funders, stakeholders, and policymakers; structured way of obtaining information; informative and intuitive quantitative outputs; studying of the level of agreement over each proposed research idea; and independent scoring of many experts which minimises the influence of individuals on the rest of the group [28,29]. Relevant stakeholders, such as patients, caregivers, and support groups are engaged in the process from an early stage, ensuring their input and investment in the outcomes [30–32] (**Figure 1**).

### Establishment of management and consultation group

To guide this research prioritisation exercise, we established a Management and Consultation Group (MCG) affiliated with the International Society of Global Health (ISoGH), which previously used the CHNRI method in 2022 to set research priorities to address the burden of COVID-19 in LMICs [26]. The ISoGH is a not-for-profit professional society based in Edinburgh, United Kingdom, actively engaged in setting global health research priorities, fostering health research capacity in LMICs, and providing open-access platforms for knowledge dissemination. We developed a protocol to guide this process in accordance with the revised guidelines for the CHNRI method application [27,28,30–32]. The MCG defined the objectives and context of the prioritisation exercise, and also coordinated various stages of the priority-setting exercise, including the definition of final criteria, development of a scoring approach, establishment of timelines, and invitation of relevant experts. This collaborative effort led to the establishment of a structured and systematic process for setting research priorities.

### Invitation of experts

The MCG invited ISoGH members from more than 100 countries worldwide to participate in this exercise if they felt that they had sufficient expertise. In the first phase, e-mails were sent to all ISoGH members and affiliates (nearly 1000 persons), soliciting their participation with details of the objectives and context of the exercise. During the second phase, we asked all members who declared both their interest and expertise to generate a minimum of three research ideas they deemed to be ‘research priorities for global pandemic preparedness in the post-COVID-19 era’; we received responses from 61 of ISoGH members. The MCG then scrutinised the submitted research ideas, eliminated duplicates, ensured that the phrasing of each idea permitted scoring against pre-defined criteria, and that they represented all key areas. This process yielded a consolidated list of 163 unique research ideas with considerable diversity in both research type and area. We then organised this list into seven sub-themes: Improving health system capacity and resilience (30 ideas); enhancing surveillance, monitoring, and evaluation (37 ideas); improving risk communication and health promotion (25 ideas); fostering policy, planning, and decision-making (32 ideas); improving global coordination, collaboration and partnerships (15 ideas); promoting equitable access (20 ideas); and fostering innovations and new technologies (21 ideas). As some ideas were listed under multiple subthemes, the overall number in this breakdown exceeds 163.

In the third phase, we re-invited ISoGH members to systematically score these ideas using the five pre-agreed priority-setting criteria: Feasibility and answerability; impact on burden reduction; potential for a paradigm shift; potential for translation and implementation; and equity. They scored the ideas based on four response options: 0 (unlikely to meet the criterion); 1 (likely to meet the criterion); left blank if the expert felt inadequately informed to make a judgment; or 0.5 if the expert possessed sufficient knowledge on the topic, but remained uncertain (although this practice was generally discouraged). The scoring was conducted without weighting or adjustments.

### Research context and criteria

We defined the context and the scoring criteria in line with recommendations from the previous exercises and guidelines [28]. Here, we defined the geographic context as ‘the whole globe’; the timeframe for expecting proposed research ideas as ‘long-term,’ considering the enduring impact of the COVID-19 pandemic and the lasting nature of pandemic preparedness initiatives; and the age group of people affected by COVID-19 was defined as ‘all ages,’ while the target population included ‘all populations.’

MCG members carefully considered the context of this exercise according to the CHNRI framework and revised the criteria most frequently employed in prior CHNRI exercises. Based on the context of this exercise and further consultations, they agreed upon five independent criteria for this exercise, which were then used to discriminate between the many proposed research ideas:

1. Feasibility and answerability – ‘Would you say that the proposed research would likely be feasible and successful in reaching the proposed endpoint?’
2. Potential for burden reduction – ‘Would you say that this research has a potential to markedly reduce the impact of a new pandemic on patients, caregivers and society (especially in LMICs?)’
3. Potential for a paradigm shift – ‘Would you say that this research is likely to result in a “paradigm shift” that could change and improve our current approaches to the problem of pandemic preparedness?’
4. Potential for translation and implementation – ‘To the best of your knowledge and experience, would you say that the proposed research would likely lead to practical application, implementation of new knowledge and/or be deliverable at scale?’
5. Impact on equity – ‘Would you say that the proposed research would be likely to improve equity among the sufferers affected by the new pandemic, their carers, and in the society as a whole?’

### Data analysis

Based on the input from the expert scores, the MCG members generated intermediate criterion-specific scores (CSS) by calculating the mean of individual scores for each research idea (across each criterion) received from all experts. All CSS ranged from 0% to 100%. We first calculated the research priority scores (RPS) for every idea according to each criterion and then combined the criterion-specific scores into overall RPS scores. We used bootstrapping (n = 1000) to compute the 95% confidence intervals (CIs). To provide insights into the level of agreement among the scorers, an improved version of the average expert agreement score based on information theory (AEA-e) was proposed, defined as the exponential of the negative entropy. Entropy is a widely used information criterion to quantify uncertainty; in this case, higher entropy implied greater uncertainty and less agreement. In contrast to the original AEA, which only used the most frequent score class for calculation, the AEA-e metric considers all score classes simultaneously, offering a robust theoretical guided interpretation. It had a lower bound of 1/3 and an upper bound of 1, and is calculated per the following formula:

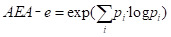

To assess the diversity of expert scoring, we performed hierarchical clustering on both the overall scores and those within each criterion. All analyses were conducted using Python, version 3.7.1 (Python Software Foundation, Wilmington, DE, USA).

### The use of ChatGPT

We prompted ChatGPT with a question to ‘please define and list, in order of importance, the greatest research priorities for global pandemic preparedness’ on 28 July 2023.

## RESULTS

### Research priority scores and expert agreement

**Figure 2:**
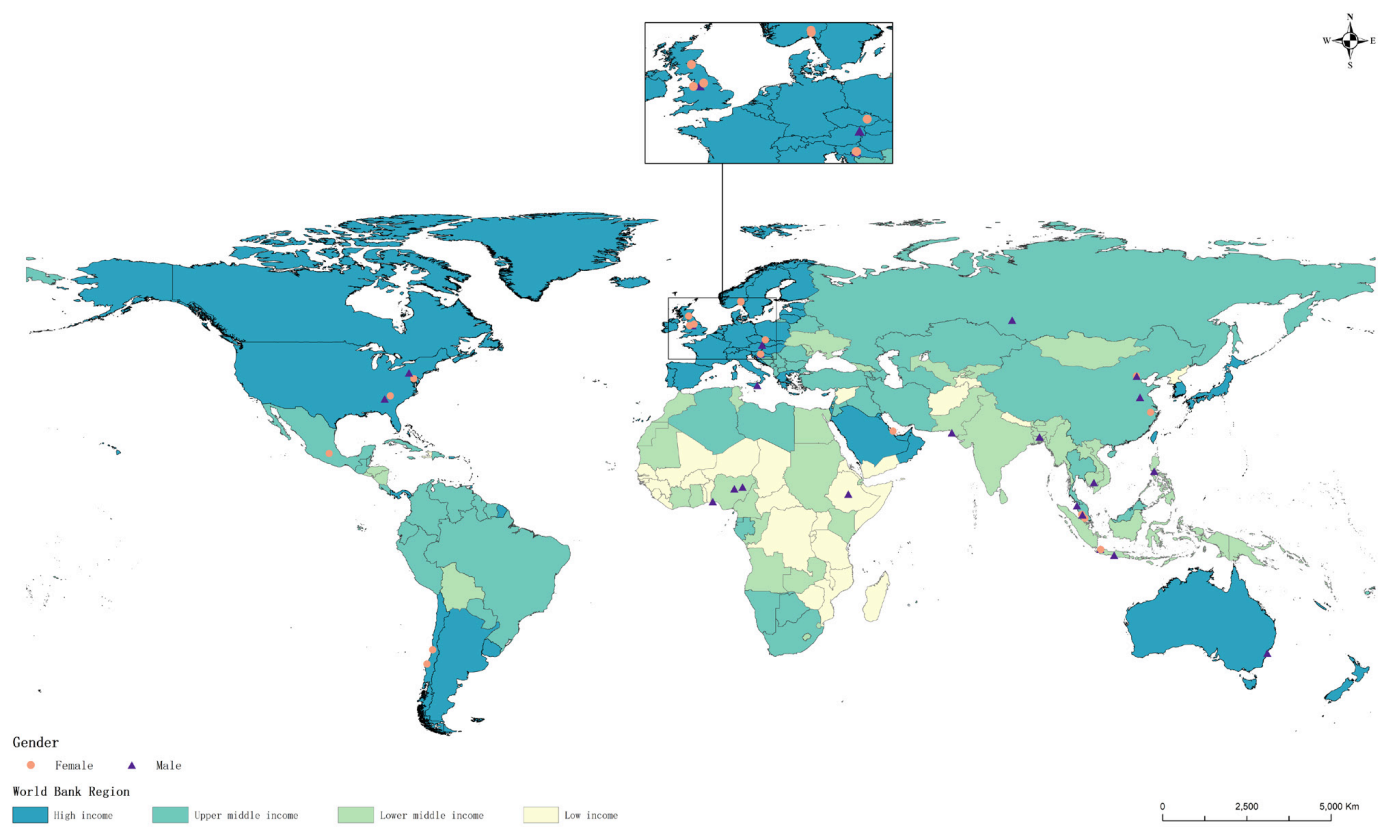
The geographic distribution of contributing scorers (n = 42).

A total of 42 ISoGH members scored the ideas; their brief information and originating countries are presented in **Figure 2** and Table S1 in the **Online Supplementary Document**.

The overall RPS for the 163 research ideas ranged from 0.317 (95% CI = 0.255–0.387) to 0.860 (95% CI = 0.805–0.900), with a median of 0.656. while the AEA-e varied from 0.414 (95% CI = 0.382–0.439) to 0.626 (95% CI = 0.558–0.673), with a median of 0.476 (Table S2 in the **Online Supplementary Document**). There was a high degree of agreement among experts on the most highly ranked research ideas, with the AEA-e decreasing with decreasing RPS (r = 0.768; *P* < 0.001). No discernible clustering patterns emerged in the overall scoring or across individual criteria (Figures S1–6 in the **Online Supplementary Document**), reflecting the diversity among participating experts and precluding the undue influence of any particular subgroups.

### Overall top-ranked priorities

Four (1st, 4th, 8th, 10th) of the top 10 research ideas prioritised enhancing health systems’ capacity and resilience. Approximately one-third (six, including two of the 10 highest scoring ideas) of the 20 top-ranked research priorities involved subtheme 6: ‘Promoting equitable access’ (**Table 1**). The top three research ideas were aimed at identifying effective strategies to strengthen pandemic preparedness and response in LMICs. The first (RPS = 0.860; 95% CI = 0.805–0.900, AEA-e = 0.602; 95% CI = 0.536–0.675) focussed on exploring effective methods to scale up vaccine and medicine production in LMICs, such as establishing regional vaccine hubs, promoting technical transfer, and providing intensive human resource training. The second-ranked idea (RPS = 0.838; 95% CI = 0.783–0.884, AEA-e = 0.626; 95% CI = 0.558–0.673) aimed to identify effective strategies for the WHO and other international organizations to enhance support for improving pandemic preparedness in resource-limited settings. The third-ranked idea (RPS = 0.834; 95% CI = 0.778–0.883, AEA-e = 0.610; 95% CI = 0.546–0.665) sought effective approaches to advance the timeliness and accuracy of pandemic surveillance, monitoring, and evaluation.

**Table 1:** The 20 highest-ranked research ideas, according to their overall RPS and AEA-e

			RPS (95% CI)						
**Rank**	**Research idea**	Subtheme*	**Feasibility and answerability**	**Impact on burden reduction**	**Potential for paradigm shift**	**Potential for translation and implementation**	**Impact on equity**	**Overall**	**Overall AEA-e (95% CI)**
1	Identifying effective strategies to scale up the production of vaccines and medicines in LMICs (e.g. regional vaccine hubs, technical transfer, human resource training)	Subtheme 1	0.878 (0.756–0.951)	0.890 (0.768–0.963)	0.744 (0.590–0.859)	0.862 (0.725–0.950)	0.923 (0.795–0.974)	0.860 (0.805–0.900)	0.602 (0.536–0.675)
2	Identifying effective strategies for the World Health Organization and other international organisations to promote their support for pandemic preparedness in LMICs	Subtheme 5	0.893 (0.774–0.964)	0.857 (0.714–0.929)	0.738 (0.595–0.857)	0.750 (0.600–0.875)	0.951 (0.829–1.000)	0.838 (0.783–0.884)	0.626 (0.558–0.673)
3	Identifying effective approaches to improve timeliness and accuracy of pandemic surveillance, monitoring, and evaluation	Subtheme 2	0.902 (0.780–0.976)	0.905 (0.786–0.976)	0.774 (0.631–0.881)	0.888 (0.750–0.962)	0.700 (0.550–0.825)	0.834 (0.778–0.883)	0.610 (0.546–0.665)
4	Evaluate approaches to sustain supply chains and avoid depletion of medical stocks during rapid surge of cases	Subtheme 1; Subtheme 4	0.854 (0.732–0.939)	0.902 (0.780–0.976)	0.732 (0.585–0.854)	0.878 (0.756–0.951)	0.750 (0.600–0.875)	0.824 (0.765–0.870)	0.600 (0.543–0.653)
5	Summarising the key lessons learnt from the COVID-19 pandemic at a local, national, and international level to improve preparedness for future pandemics	Subtheme 4	0.929 (0.810–0.976)	0.881 (0.762–0.952)	0.683 (0.537–0.817)	0.821 (0.690–0.917)	0.775 (0.625–0.900)	0.819 (0.763–0.867)	0.586 (0.526–0.645)
6	Studying the local contexts in LMICs to develop equitable community-based interventions that can mitigate the effects of the pandemic	Subtheme 6	0.838 (0.688–0.925)	0.951 (0.829–1.000)	0.683 (0.537–0.805)	0.731 (0.577–0.846)	0.866 (0.732–0.951)	0.814 (0.757–0.864)	0.581 (0.520–0.639)
7	Identify gaps in planning and implementation of the programs to combat pandemics in LMICs	Subtheme 4	0.938 (0.825–0.988)	0.875 (0.750–0.950)	0.692 (0.538–0.821)	0.788 (0.649–0.888)	0.744 (0.590–0.846)	0.808 (0.750–0.856)	0.566 (0.510–0.630)
8	Developing effective approaches to improve and maintain supply chains (donation and purchase, distribution, continuous supply, local production) of medicines and all other goods	Subtheme 1	0.903 (0.778–0.972)	0.821 (0.667–0.923)	0.692 (0.538–0.821)	0.821 (0.679–0.910)	0.806 (0.645–0.917)	0.807 (0.749–0.8600)	0.573 (0.513–0.629)
9	Developing approaches to improve availability and timely roll-out of vaccines and medicines rapidly developed to tackle new pandemics	Subtheme 6	0.869 (0.738–0.952)	0.940 (0.833–0.988)	0.634 (0.463–0.780)	0.762 (0.619–0.869)	0.805 (0.659–0.902)	0.803 (0.745–0.851)	0.564 (0.510–0.623)
10	Explore approaches to strengthening the primary care (as the most accessible and equitable point of care) in pandemic response, to include vaccination, surveillance, diagnosis and treatment	Subtheme 1	0.810 (0.667–0.905)	0.952 (0.838–1.000)	0.667 (0.500–0.810)	0.762 (0.619–0.869)	0.810 (0.667–0.905)	0.800 (0.743–0.850)	0.580 (0.526–0.634)
11	Studying barriers to equal access to diagnosis, consultation, and treatments for disadvantaged groups (including people with disabilities, children, minorities, etc.)	Subtheme 6	0.890 (0.768–0.963)	0.700 (0.550–0.825)	0.671 (0.512–0.805)	0.750 (0.600–0.862)	0.854 (0.720–0.939)	0.773 (0.717–0.825)	0.513 (0.463–0.565)
12	Identify strategies that can be used to address the mental health burden of a pandemic in low mental health resource settings	Subtheme 6	0.829 (0.695–0.915)	0.780 (0.634–0.878)	0.622 (0.476–0.768)	0.793 (0.646–0.890)	0.829 (0.683–0.927)	0.771 (0.710–0.822)	0.525 (0.479–0.581)
13	Developing an accurate and reliable pandemic preparedness index to support LMICs in their pandemic preparedness planning	Subtheme 4	0.825 (0.675–0.925)	0.838 (0.700–0.925)	0.712 (0.550–0.838)	0.762 (0.612–0.875)	0.675 (0.525–0.800)	0.762 (0.700–0.815)	0.542 (0.489–0.588)
14	Identifying the challenges to supplying and delivering vaccines safely and efficiently in LMICs	Subtheme 6	0.857 (0.714–0.929)	0.810 (0.667–0.905)	0.588 (0.438–0.725)	0.702 (0.548–0.833)	0.833 (0.690–0.929)	0.760 (0.695–0.812)	0.550 (0.503–0.597)
15	Designing behavioural strategies to improve public compliance and buy-in for infection control measures	Subtheme 3	0.789 (0.632–0.895)	0.872 (0.744–0.949)	0.763 (0.605–0.868)	0.737 (0.579–0.868)	0.641 (0.487–0.795)	0.760 (0.693–0.818)	0.577 (0.542–0.617)
16	Identifying effective strategies for high income countries to expand their funding support to LMICs in improving pandemic preparedness	Subtheme 5	0.810 (0.667–0.905)	0.881 (0.738–0.952)	0.631 (0.476–0.762)	0.549 (0.390–0.695)	0.915 (0.793–0.976)	0.757 (0.700–0.810)	0.539 (0.490–0.584)
17	Evaluate approaches to improve technology capacity of vaccine development and equality of vaccine deployment in LMICs	Subtheme 6; Subtheme 7	0.774 (0.619–0.881)	0.829 (0.683–0.927)	0.643 (0.476–0.786)	0.762 (0.619–0.881)	0.774 (0.631–0.881)	0.756 (0.694–0.809)	0.548 (0.500–0.596)
18	Identifying the most effective data sharing and data use practices for epidemic surveillance for the emerging and re-emerging infections in LMICs	Subtheme 2	0.862 (0.725–0.950)	0.846 (0.692–0.923)	0.763 (0.605–0.868)	0.679 (0.526–0.808)	0.618 (0.461–0.763)	0.755 (0.691–0.809)	0.536 (0.483–0.583)
19	Developing a framework for mitigating a shortage of medical products/consumables and monitoring and notification of shortages	Subtheme 1	0.900 (0.775–0.975)	0.795 (0.641–0.897)	0.600 (0.425–0.750)	0.769 (0.615–0.872)	0.684 (0.526–0.816)	0.750 (0.684–0.806)	0.570 (0.538–0.616)
20	Assessing the effectiveness of Early Warning Outbreak Recognition System based on the One Health approach	Subtheme 2	0.857 (0.714–0.943)	0.794 (0.647–0.912)	0.706 (0.529–0.853)	0.803 (0.636–0.909)	0.574 (0.397–0.721)	0.747 (0.676–0.806)	0.538 (0.483–0.590)

AEA-e – average expert agreement score based entropy, CI – confidence interval, LMIC – low- and middle-income country, RPS – research priority scores,

*Subtheme 1: Improving health system capacity and resilience. Subtheme 2: Enhancing surveillance, monitoring and evaluation. Subtheme 3: Improving risk communication and health promotion. Subtheme 4: Fostering policy, planning and decision making. Subtheme 5: Improving coordination and collaboration. Subtheme 6: Promoting equitable access. Subtheme 7: Fostering innovations and new technologies.

The 4th and 7th priority ideas aimed to evaluate approaches for sustaining supply chains and identifying gaps in planning and implementing health programs against pandemics in LMICs. The 5th priority idea focused on summarising key lessons from COVID-19 at the local, national, and global levels to enhance future pandemic preparedness. The 6th research priority idea focussed on studying local contexts in LMICs to develop equitable community-based interventions. The 8th and 9th ideas targeted the development of approaches to ensure the timely availability and supply chains of medicines and other essential goods. The 10th research priority investigated methods to strengthen primary care in pandemic response, including vaccination, surveillance, diagnosis, and treatment.

Other topics among the 20 highest-scoring ideas were specific to studying barriers to equal access to diagnosis and treatment for disadvantaged groups, identifying strategies to address the mental health burden in low-resource settings, and developing a reliable pandemic preparedness index for LMICs. The importance of identifying challenges to supplying and delivering vaccines safely and efficiently in LMICs and designing behavioural strategies to improve public compliance with infection control measures was also highlighted. The remaining ideas (ranked 16th to 20th) focussed on enhancing pandemic preparedness in LMICs through strategies like increased funding from HICs; improved vaccine development technology and equity; effective data sharing practices for epidemic surveillance; a framework for mitigating and monitoring medical product shortages; and an assessment of the Early Warning Outbreak Recognition System based on the One Health approach.

### Top-ranked priorities within specific priority-setting criteria

The three highest-scoring research ideas according to each criterion are presented in **Table 2**. When research ideas were assessed and ranked according to the criterion ‘Feasibility and answerability,’ the top two focussed on uncovering gaps in pandemic response programs in LMICs and summarising key lessons from the COVID-19 pandemic to improve future preparedness at all levels. One notable idea that did not appear in the top 20 research priorities, but scored the third in this criterion alone, was analysing if the WHO Checklist for pandemic preparedness is a valid and useful tool for LMICs.

**Table 2:** The three highest-ranked research ideas for each of the predefined priority-setting criteria

Overall rank	Subtheme*	Research idea	RPS (95% CI)	AEA-e (95% CI)
**Feasibility and answerability**				
7	Subtheme 4	Identify gaps in planning and implementation of the programs to combat pandemics in LMICs.	0.938 (0.825–0.988)	0.730 (0.526–0.890)
5	Subtheme 4	Summarising the key lessons learnt from the COVID-19 pandemic at a local, national, and international level to improve preparedness for future pandemics.	0.929 (0.810–0.976)	0.773 (0.615–0.894)
117	Subtheme 2	Analysing if the World Health Organization Checklist for pandemic preparedness is a valid and useful tool for LMICs.	0.927 (0.805–0.976)	0.770 (0.633–0.892)
**Impact on burden reduction**				
10	Subtheme 1	Explore approaches to strengthening the primary care (as the most accessible and equitable point of care) in pandemic response, to include vaccination, surveillance, diagnosis and treatment.	0.952 (0.838–1.000)	0.826 (0.637–1.000)
6	Subtheme 6	Studying the local contexts in LMICs to develop equitable community-based interventions that can mitigate the effects of the pandemic.	0.951 (0.829–1.000)	0.823 (0.627–1.000)
9	Subtheme 6	Developing approaches to improve availability and timely roll-out of vaccines and medicines rapidly developed to tackle new pandemics.	0.940 (0.833–0.988)	0.739 (0.539–0.894)
**Potential for paradigm shift**				
3	Subtheme 2	Identifying effective approaches to improve timeliness and accuracy of pandemic surveillance, monitoring and evaluation.	0.774 (0.631–0.881)	0.535 (0.415–0.637)
15	Subtheme 3	Designing behavioural strategies to improve public compliance and buy-in for infection control measures.	0.763 (0.605–0.868)	0.578 (0.511–0.714)
18	Subtheme 2	Identifying the most effective data sharing and data use practices for epidemic surveillance for the emerging and re-emerging infections in LMICs.	0.763 (0.605–0.868)	0.578 (0.511–0.714)
**Potential for translation and implementation**				
3	Subtheme 2	Identifying effective approaches to improve timeliness and accuracy of pandemic surveillance, monitoring and evaluation.	0.888 (0.750–0.962)	0.644 (0.482–0.820)
4	Subtheme 1; Subtheme 4	Evaluate approaches to sustain supply chains and avoid depletion of medical stocks during rapid surge of cases.	0.878 (0.756–0.951)	0.690 (0.574–0.823)
1	Subtheme 1	Identifying effective strategies to scale up the production of vaccines and medicines in LMICs (e.g. regional vaccine hubs, technical transfer, human resource training).	0.862 (0.725–0.950)	0.612 (0.450–0.766)
**Impact on equity**				
2	Subtheme 5	Identifying effective strategies for the World Health Organization and other international organisations to promote their support for pandemic preparedness in LMICs.	0.951 (0.829–1.000)	0.823 (0.659–1.000)
1	Subtheme 1	Identifying effective strategies to scale up the production of vaccines and medicines in LMICs (e.g. regional vaccine hubs, technical transfer, human resource training).	0.923 (0.795–0.974)	0.762 (0.602–0.888)
16	Subtheme 5	Identifying effective strategies for high income countries to expand their funding support to LMICs in improving pandemic preparedness.	0.915 (0.793–0.976)	0.688 (0.526–0.823)

AEA-e – average expert agreement score-based entropy, CI – confidence interval, LMIC – low- and middle-income country, RPS – research priority scores

*Subtheme 1: Improving health system capacity and resilience. Subtheme 2: Enhancing surveillance, monitoring and evaluation. Subtheme 3: Improving risk communication and health promotion. Subtheme 4: Fostering policy, planning and decision making. Subtheme 5: Improving coordination and collaboration. Subtheme 6: Promoting equitable access. Subtheme 7: Fostering innovations and new technologies.

The criterion ‘Impact on burden reduction’ was ranked highly for three research ideas that also made it into the overall top 10: Enhancing primary care’s role in pandemic response; examining local contexts in LMICs for equitable, community-based pandemic interventions; and developing strategies to ensure rapid availability and distribution of vaccines and medicines during new pandemics.

Regarding the criterion ‘Potential for paradigm shift,’ the research to identify effective approaches to improve timeliness and accuracy of pandemic surveillance, monitoring, and evaluation ranked first for this criterion and third in the overall assessment. Simultaneously, developing behavioural strategies to enhance public compliance with infection control measures and determining the best data-sharing and utilisation practices for pandemic surveillance in LMICs were also seen as potentially leading to a paradigm shift.

For the criterion ‘Potential for translation and implementation,’ the three highest-scoring research ideas were also within the top five on the overall list and included enhancing the timeliness and accuracy of pandemic surveillance, monitoring, and evaluation; evaluating methods to maintain supply chains and prevent medical stock depletion during rapid case surges; and finding strategies to increase vaccine and medicine production in LMICs.

Finally, the top two scoring ideas from the ‘Impact on equity’ criterion were discovering effective strategies for international organisations (like the WHO) to bolster pandemic preparedness in LMICs and identifying ways to increase vaccine and medicine production in LMICs. These were closely followed by the research idea of determining strategies for HICs to enhance financial support to LMICs for improved pandemic preparedness, which was predicted to have a high likelihood of improving impact on equity.

### ChatGPT’s output

The output from ChatGPT is presented in **Box 1**. Clearly, the rapid development and accelerated approval and roll-out of vaccines and medicines (antivirals) are at the two top spots – which corresponds to the top priority identified by the ISoGH members. Surveillance and early detection ranked 3rd in both exercises, which was another striking similarity. However, the 2nd priority of the ISoGH exercise – on the improved role for the WHO and other organisations – did not emerge as prominently in ChatGPT’s output, although it was broadly covered as its 8th priority.

Box 1 **The output from ChatGPT, version 3.5., when prompted to ‘please define and list, in order of importance, the greatest research priorities for global pandemic preparedness’(on 28 July 2023.**

*Global pandemic preparedness is a critical area of research that requires ongoing efforts and continuous improvement. As of my last update in September 2021, the following list represents the greatest research priorities for pandemic preparedness, listed in order of importance:*

*1. Vaccine Development and Accelerated Approval: Developing effective vaccines against a wide range of pathogens and streamlining the approval process are vital to respond rapidly to emerging infectious diseases. Research should focus on novel vaccine platforms, universal vaccines, and efficient production and distribution methods.*

*2. Antiviral Therapeutics: Developing antiviral drugs that can effectively treat a broad spectrum of viruses is crucial. Research should target viral replication mechanisms and identify potential drug targets to create effective therapeutics.*

*3. Surveillance and Early Detection Systems: Improving global surveillance networks, including real-time data sharing, early warning systems, and advanced diagnostic technologies, can significantly enhance the ability to detect and respond quickly to outbreaks.*

*4. Public Health Infrastructure and Response: Strengthening public health systems globally is essential. Research should focus on optimizing response protocols, communication strategies, and resource allocation during pandemics.*

*5. One Health Approach: Understanding the interconnectedness of human, animal, and environmental health is vital for early detection and prevention of zoonotic diseases. Research should emphasize the collaboration between human and veterinary medicine.*

*6. Behavioral and Social Sciences: Investigating human behavior during pandemics, including risk perception, adherence to public health measures, and effective communication strategies, is essential to tailor interventions and improve compliance.*

*7. Pathogen Characterization and Genomics: Rapidly identifying and characterizing pathogens during outbreaks is crucial for devising targeted response strategies. Genomic research can aid in tracing the origin, spread, and evolution of pathogens.*

*8. Global Coordination and Governance: Researching effective global coordination and governance structures can improve international collaboration, resource sharing, and coordinated responses during pandemics.*

*9. Medical Supply Chain and Stockpiling: Researching efficient and resilient medical supply chains, including the stockpiling of essential medical equipment and supplies, can help ensure sufficient resources during emergencies.*

*10. Vulnerable Populations: Understanding the unique challenges faced by vulnerable populations, such as the elderly, immunocompromised individuals, and marginalized communities, is crucial for developing inclusive and equitable pandemic response strategies.*

*11. Risk Communication and Misinformation: Researching effective risk communication strategies and combatting misinformation can improve public understanding, trust in public health institutions, and adherence to preventive measures.*

*12. Innovative Technologies: Exploring and developing innovative technologies such as AI, big data analytics, telemedicine, and remote monitoring can enhance pandemic response capabilities.*

*13. Ethical Considerations: Research should delve into the ethical implications of pandemic preparedness and response, including issues of equity, privacy, and resource allocation.*

*14. Economic and Social Impact Analysis: Understanding the economic and social impacts of pandemics can inform policies that mitigate the negative effects on livelihoods and societies.*

*These priorities are not exhaustive, and the field of pandemic preparedness is continually evolving. Continuous research, international collaboration, and investment in public health infrastructure are necessary to effectively address future pandemics and protect global health.*

Next identified research priorities in the CHNRI-based exercise were dominated by concerns over supply chains and health systems’ capacity, which is also broadly covered by the ChatGPT’s 4th priority, suggesting another similar and mutually supportive ranking. Then, ChatGPT ranked the ‘One health’ approach and tracing pathogens genomically across species as the 5th and the 7th priority, while in the CHNRI exercise this was captured by the priority questions ranked 20th (specifically) and 5th (more generally).

Both exercises placed value to behavioural and social sciences in the pandemic – ChatGPT ranked this as the 6th priority, while it was encompassed by the ideas ranked 13th and 15th in the CHNRI exercise. Furthermore, ChatGPT identified the challenges with the supply chain as the 9th priority, while this research theme was more prominent in the CHNRI exercise (ranked 4th, 8th, 14th, and 19th). The 10th priority identified by ChatGPT – ‘Vulnerable populations’ – was given higher prominence in the CHNRI exercise, where this was an important element of the questions ranked 6th, 9th, 11th, 12th and 16th. The priorities ranked 11th to 13th by ChatGPT (e.g. risk communication and misinformation, innovative technologies, and ethical considerations) were also captured in the top 20 CHNRI priority questions. The one specific research priority which ChatGPT still ranked highly, while it was absent from the top 20 CHNRI ideas was ‘economic and social impact analysis.’

## DISCUSSION

### Main findings

As the pandemic evolved, and with the recent WHO determination that the pandemic is no longer classified as a Public Health Emergency of International Concern, it has become crucial to understand how LMICs can enhance their pandemic preparedness for future emergencies, especially in the context of scarce resources and other health systems challenges. In this study, four of the top ten ideas (1st, 5th, 9th, 10th) prioritised optimising production and supply chains for vaccines and medicines, preventing depletion of medical stocks during a surge, maintaining supply chain efficiency, and strengthening primary care delivery infrastructure for effective pandemic response. Three of the top ten ideas were aimed at understanding how to improve planning and response to future pandemics by learning from COVID-19 experiences in sustaining supply chains, avoiding shortages, and addressing related gaps in LMICs. Developing locally-appropriate, equitable community-based interventions (7th), improving availability of new vaccines and treatments during pandemics (8th), identifying effective strategies for international organizations to support strengthening pandemic preparedness in LMICs (2nd), and effective approaches to improve timeliness and accuracy of pandemic surveillance, monitoring and evaluation (3rd) were also among the most highly scoring priorities.

### Strengths and limitations

The primary strength of this research priority-setting exercise lies in the utilisation of the CHNRI approach, which is transparent, replicable, and feasible to apply. This methodology has been refined and enhanced through numerous implementations in recent years [21,27,28]. In our study, we made further methodological improvements – we calculated the 95% CIs of RPS and proposed the use of an improved AEA score, the AEA-e, which is the exponential of the negative entropy. Rather than using the most frequent score class for calculation in the conventional manner, AEA-e takes all score classes into consideration simultaneously and offers a theoretically guided interpretation. The output of this study is intuitive and easily comprehended. Moreover, the comparison to ChatGPT’s output further strengthened the main conclusions.

Additionally, previous experiences and statistical simulations have indicated considerable convergence of collective expert opinions when the number of scoring experts reaches 40 or more (our study had 42 scoring experts), ensuring stability and replicability of the final rankings according to CHNRI methods [21,27]. Consistent with this, we observed a high degree of agreement among experts on the most highly ranked research ideas based on AEA-e values [22,26]. Again, ChatGPT’s output helped with confirming the identified priorities. Moreover, we detected no discernible clustering patterns in either the overall scoring or individual criteria. This diversity – also evidenced by representation of respondents from many countries and world regions – added robustness to our results and provided a wide range of perspectives within the scientific community.

A major limitation to consider when interpreting our findings is that the participating experts were identified from the ISoGH, rather than from the broader community involved in the COVID-19 pandemic response or pandemic preparedness. However, our goal here was two-fold. First, by conducting this CHNRI exercise within the ISoGH, we were able to mobilise global health experts from LMICs. Second, COVID-19 pandemic response was challenged by an evolving lack of trust toward official institutions involved in response; therefore, adding a voice from an independent, not-for-profit organisation to the public debate on priorities for pandemic preparedness might represent a useful additional guidance to policymakers globally. Largely, while countries may need to contextualise their research priorities, our findings could serve as an independent external guide for such exercises. Moreover, experts were allowed, albeit discouraged, to employ a ‘0.5′ response in cases where they possessed sufficient knowledge on the topic but remained uncertain. This might have contributed to a ‘regression to the mean’ effect in the final distribution of the overall RPS. Careful consideration of this potential impact is necessary to guarantee the validity and robustness of those findings.

While representativeness is difficult to ensure with any approach, the CHNRI method relies on expert-sourcing to mitigate these effects. It produces the best results when scorers provide their private, independent views and when there is a diversity of opinions. Previous research has shown that rankings of research ideas by independent scorers with some topic familiarity become stable after about 40 participants, with very little change in rankings upon addition of more expert scorers [21,27]. It is positive for this exercise that we detected no clustering among these global health experts, largely ensuring that the resulting priorities reflect collective rather than individual views.

### Findings in the context of the literature

Research efforts focusing on effective strategies to scale up the production of vaccines and medicines in LMICs were found to be paramount, with the highest overall RPS of 0.863 (95% CI = 0.810–0.905). This research idea also ranked highly (2nd) in both the criteria of ‘potential for translation and implementation’ and ‘impact on equity.’ The COVID-19 pandemic has starkly exposed disparities in access to vaccines and medicines across socioeconomic levels, regions, and countries [33,34]. As such, it is unsurprising that addressing this pressing issue by scaling up production, enhancing self-sufficiency and ensuring a more equitable distribution of essential health care resources is considered a priority. However, many experts appear to understand the contextual challenges in the feasibility and/or scale-up of production of vaccines and medicines in many LMICs [34], which reflects in the ranking of this idea as only 16th for the ‘feasibility and answerability’ criterion.

Overall, identifying effective strategies for the WHO and other international organisations to promote their support for pandemic preparedness in LMICs was ranked 2nd, and it was the leading one out of the 163 proposed ideas in the criterion of ‘impact on equity.’ In this pandemic, the role of the WHO and other international organisations appear to have been under-emphasised and rarely exploited at country levels, despite available resources to support efforts – especially in LMICs – such as financial support, technical assistance, sharing information, and best practices, among others [35,36]. However, many experts thought that the potential of this idea for translation and implementation may be unrealistic, which resulted in the rank 12th for that criterion. This calls for concerted efforts among global partners to work together to make international support and collaboration in the response to pandemics implementable.

The third overall priority focused on ‘identifying effective approaches to improve the timeliness and accuracy of pandemic surveillance, monitoring, and evaluation.’ This achieved top rankings under ‘potential for paradigm shift’ and ‘potential for translation and implementation.’ It also scored high in ‘feasibility and answerability’ (6th) and ‘impact on burden reduction’(4th), suggesting that effective pandemic surveillance, monitoring, and evaluation should be attainable goals, even for many LMICs. However, in less resourced settings, well-functioning surveillance, monitoring, and evaluation systems are predominantly found in urban and more affluent areas, making it difficult to maintain equity across regions within those countries [37,38]. Consequently, the ‘impact on equity’ for this priority received a lower ranking (45th).

Ideas regarded as less important by the experts in this exercise mainly encompass topics not directly linked to immediate pandemic response or critical health care concerns. Some ideas focused on broader global challenges, such as Sustainable Development Goals, climate change, and online education. Although important, they may not have immediate and/or direct implications for pandemic preparedness in many settings. Some ideas, such as describing pandemic waves, may have been seen as already sufficiently done. Other ideas, such as those related to career satisfaction, chatbots in primary health care, or alternative medicine practitioners, could be perceived as unconventional, futuristic, or overshadowed by more urgent research areas.

### Findings in the context of the ChatGPT’s output

The comparison of the results of the CHNRI process to the ChatGPT’s output highlighted many striking similarities and was useful as an additional reassurance. It seems that both collective human opinion and knowledge, harnessed through the CHNRI process, and ChatGPT – which, essentially, also harnesses human collective knowledge – came to very similar conclusions. The key differences between the two are that ChatGPT’s output seems very theoretical, identifying and listing broad research areas that should be prioritized from the existing knowledge. The outputs of the CHNRI process are more specific and nuanced, and are also clearly influenced by the recent experiences with the COVID-19 pandemic.

This study is the very first, to the best of our knowledge, to present both the results of human collective opinion and human collective knowledge and compare them directly. We found this unexpected opportunity fascinating: It was reassuring that the obtained results were eventually quite similar through both approaches. It is always expected from the CHNRI exercises to answer the question on the representativeness of the collective opinion of the particular group involved. Therefore, ‘validation’ of the output through another source – in this case, ChatGPT – is a welcome opportunity. In this, we do not imply that ChatGPT or any presently available AI tool should be considered a ‘gold standard’ for any priority-setting exercises based on human collective opinion – we need a lot more experience with their use before we can draw firmer conclusions.

### Research, funding, and policy implications

This study represents one of the first systematic approaches to identifying research priorities for pandemic preparedness following the COVID-19 outbreak. This is particularly important for LMICs, as they must navigate the challenges of the post-COVID-19 era and address the weakened and disrupted health systems, or even society, with resource constraints [5,39,40]. Moreover, these research priorities hold broader implications for the global health research community, as the lessons learned from LMICs can inform strategies for pandemic preparedness and response in other settings.

However, it is essential to acknowledge that the research priorities established in this study were set within the overarching context of LMICs. Due to the heterogeneity among health care systems, governance structures, financing models, sociocultural practices, and the consequences of COVID-19 across different LMICs, the application of this research priority-setting exercise may vary between settings.

The CHNRI method is not prescriptive. Its main purpose is to expose strengths and weaknesses of a very large number of research ideas. The users can pick research ideas from the entire list and build a portfolio of research ideas to support. In doing so, they will be guided by a collective optimism of the reasonably large group of experts, whose collective input will signal how each of the research ideas would satisfy each of the important criteria. In this way, the CHNRI method protects those who are making investment decisions from taking too high risks wherever there are clear concerns expressed by the experts. Still, it allows everyone to find and pick the ideas that are most feasible in their own context. To maximise the utility and effectiveness of these priorities, policymakers and researchers should consider national-, sub-national-, or even local-level contexts when developing tailored policies and programs. This contextualised approach can help ensure that interventions and strategies are appropriately adapted to suit the unique needs and challenges of specific regions, ultimately enhancing the preparedness and resilience of health care systems in the face of future pandemics.

## CONCLUSIONS

We carried out a rigorous expert consensus process to identify research priorities for global pandemic preparedness, incorporating the perspectives of numerous international experts and the ChatGPT large language model. We believe that this is an example of how to make AI tools useful to decision-makers – they can be very helpful when they ‘validate’ the human collective opinion. Very few investors are entirely comfortable with investing based on a collective opinion of any group of people. Similarly, very few are entirely comfortable with relying on AI alone. However, when combined, the two approaches can provide reassurance of each other’s output.

While the leading priorities identified focused on traditional research and development, many were related to operational and/or implementation research which perhaps reflects the main challenges in many countries. Addressing these priorities, along with introducing improvements in health planning, undertaking equitable community-based interventions, and expanding the capacity of primary health care, is vital for better pandemic preparedness and response in many settings. As the COVID-19 pandemic has ceased to be classified as a global health emergency, we advocate urgent attention to the reinforcement and improvement of global pandemic preparedness strategies.

## Additional material


Online Supplementary Document.
