# Supplementary material for: Setting research priorities for global pandemic preparedness: An international consensus and comparison with ChatGPT’s output
Source: J Glob Health. 2024 Feb 16;14:04054. doi: 10.7189/jogh.14.04054 (PMC10869134; doi:10.7189/jogh.14.04054)
Supplement: Online Supplementary Document. [file jogh-14-04054-s001.pdf]

Supplementary Appendix

Table S1. Characteristics of experts contributing to this CHNRI exercise

Table S2. Full list of the 163 research priorities ranked by their overall RPS
Note: 95% CI, 95% confidence interval; RPS, Research Priority Scores; AEA-e, average expert agreement score based entropy; Subtheme 1: improving health system capacity and resilience; Subtheme 2: enhancing surveillance, monitoring and evaluation; Subtheme 3: improving risk communication and health promotion; Subtheme 4: fostering policy, planning and decision making; Subtheme 5: improving coordination and collaboration; Subtheme 6: promoting equitable access; Subtheme 7: fostering innovations and new technologies.

Figure S1. The hierarchical clustering of all idea scores from contributing experts

Figure S2. The clustering of idea scores in the criterion-"feasibility and answerability"

Figure S3. The clustering of idea scores in the criterion-"impact on burden reduction"

Figure S4. The clustering of idea scores in the criterion-"potential for paradigm shift"

Figure S5. The clustering of idea scores in the criterion-"potential for translation and implementation"

Figure S6. The clustering of idea scores in the criterion-"impact on equity"

Table S1. Characteristics of experts contributing to this CHNRI exercise

Name	Gender	Country	Income region	Affiliation
Adams Bodomo	Male	Austria and Ghana national	High income	African Studies, University of Vienna
Emma Sacks	Female	United States	High income	Johns Hopkins Bloomberg School of Public Health
Sajjad Ali	Male	Pakistan	Lower middle income	Department of Medicine, Ziauddin Medical University
Rosnah Sutan	Female	Malaysia	Upper middle income	Universiti Kebangsaan Malaysia Medical Centre
Sandro Vento	Male	Cambodia	Lower middle income	University of Puthisastra
Elena S. Rotarou	Female	Chile	High income	Universidad San Sebastián
Mila Nu Nu Htay	Female	Malaysia	Upper middle income	Department of Community Medicine, Faculty of Medicine, Manipal University College Malaysia
Konstantin Sharov	Male	Russia	Upper middle income	Koltzov Institute of Developmental Biology of Russian Academy of Sciences
Monika Berecki	Female	Croatia	High income	School of Medicine, University of Zagreb
Rowalt Alibudbud	Male	Philippines	Lower middle income	Department of Sociology and Behavioral Sciences, De La Salle University
Sheri Bastien	Female	Norway	High income	Norwegian University of Life Sciences
Teshome Gebre	Male	Ethiopia	Low income	The Task force for Global Health
Suleiman Mshelia	Male	Nigeria	Lower middle income	Jos University Teaching Hospital
Florencia Borrescio-Higa	Female	Chile	High income	Universidad Adolfo Ibañez
Jingyi Xu	Female	China	Upper middle income	School of Health Humanities, Peking University
Christopher J. Graham	Male	United Kingdom	High income	Faculty of Biology, Medicine and Health, The

Name	Gender	Country	Income region	Affiliation
Zhi Xiang Ng	Male	Malaysia	Upper middle income	University of Manchester School of Biosciences, Faculty of Science and Engineering, University of Nottingham Malaysia
Sohaila Cheema	Female	Qatar	High income	Weill Cornell Medicine – Qatar
Yubraj Acharya	Male	United States	High income	Department of Health Policy and Administration, The Pennsylvania State University
Darien Alfa Cipta	Male	Indonesia	Lower middle income	Universitas Pelita Harapan
Michelle Parisi	Female	United States	High income	Clemson University
Yanfeng Zhang	Male	China	Upper middle income	Capital Institute of Pediatrics
Liang Wang	Male	China	Upper middle income	Guangdong Provincial People's Hospital, China
Giuseppe Lanza	Male	Italy	High income	University of Catania Oasi Research Institute-IRCCS
Chinonso Igwesi-Chidobe	Female	United Kingdom	High income	Africa University of Bradford/University of Nigeria
Wenang Supriyatiningsih	Female	Indonesia	Lower middle income	Children and Mother Health Movement Action
Dialechti Tsimpida	Female	United Kingdom	High income	Department of Public Health, Policy and Systems, The University of Liverpool
Mohammad Mainul Islam	Male	Bangladesh	Lower middle income	University of Dhaka
Peige Song	Female	China	Upper middle income	School of Public Health and Women's Hospital, Zhejiang University School of Medicine
Per Ole Iversen	Female	Norway	High income	Department of Nutrition, University of Oslo
Prince Peprah	Male	Australia	High income	Social Policy Research Centre/Centre for Primary Health Care and Equity,

Name	Gender	Country	Income region	Affiliation
Renata Riha	Female	United Kingdom	High income	University of New South Wales Royal Infirmary of Edinburgh, University of Edinburgh
Srdjan Stankov	Male	Serbia	Upper middle income	Pasteur Institute Novi Sad
Kabiru Olusegun Akinyemi	Male	Nigeria	Lower middle income	Lagos State University
Anton Glasnovic	Male	Croatia	High income	Croatian Institute for Brain Research, Zagreb University School of Medicine
Danladi Adamu Bojude	Male	Nigeria	Lower middle income	Gombe State University
Francisco Becerra-Posada	Male	United States	High income	Public Health Development Organization
Josipa Vlasac Glasnović	Female	Croatia	High income	Department of Hematology, Dubrava University Hospital
Kit Chan	Female	United Kingdom/Australia	High income	Centre for Global Health, Usher Institute, University of Edinburgh/School of Social Sciences, Monash University
Kurubaran Ganasegeran	Male	Malaysia	Upper middle income	Seberang Jaya Hospital, Ministry of Health
Lina Diaz Castro	Female	Mexico	Upper middle income	National Institute of Psychiatry Ramón de la Fuente Muñiz
Marie Buchtova	Female	Czechia	High income	Olomouc University Social Health Institute, Palacký University

Table S2. Full list of the 163 research priorities ranked by their overall RPS

Rank	Research idea	Subtheme	Overall RPS (95% CI)	Overall AEA-e (95% CI)
1	Identifying effective strategies to scale up the production of vaccines and medicines in LMICs (e.g., regional vaccine hubs, technical transfer, human resource training)	Subtheme 1	0.860 (0.805 - 0.900)	0.602 (0.536 - 0.675)
2	Identifying effective strategies for the WHO and other international organizations to promote their support for pandemic preparedness in LMICs	Subtheme 5	0.838 (0.783 - 0.884)	0.626 (0.558 - 0.673)
3	Identifying effective approaches to improve timeliness and accuracy of pandemic surveillance, monitoring and evaluation	Subtheme 2	0.834 (0.778 - 0.883)	0.610 (0.546 - 0.665)
4	Evaluate approaches to sustain supply chains and avoid depletion of medical stocks during rapid surge of cases	Subtheme 1; Subtheme 4	0.824 (0.765 - 0.870)	0.600 (0.543 - 0.653)
5	Summarizing the key lessons learnt from the COVID-19 pandemic at a local, national, and international level to improve preparedness for future pandemics	Subtheme 4	0.819 (0.763 - 0.867)	0.586 (0.526 - 0.645)
6	Studying the local contexts in LMICs to develop equitable community-based interventions that can mitigate the effects of the pandemic	Subtheme 6	0.814 (0.757 - 0.864)	0.581 (0.520 - 0.639)
7	Identify gaps in planning and implementation of the programs to combat pandemics in LMIC	Subtheme 4	0.808 (0.750 - 0.856)	0.566 (0.510 - 0.630)
8	Developing effective approaches to improve and maintain supply chains (donation and purchase, distribution, continuous supply, local production) of medicines and all other goods	Subtheme 1	0.807 (0.749 - 0.860)	0.573 (0.513 - 0.629)
9	Developing approaches to improve availability and timely roll-out of vaccines and medicines rapidly developed to tackle new pandemics	Subtheme 6	0.803 (0.745 - 0.851)	0.564 (0.510 - 0.623)
10	Explore approaches to strengthening the primary care (as the most accessible and equitable point of care) in pandemic response, to include vaccination, surveillance, diagnosis and treatment	Subtheme 1	0.800 (0.743 - 0.850)	0.580 (0.526 - 0.634)
11	Studying barriers to equal access to diagnosis, consultation, and treatments for disadvantaged groups (including people with disabilities, children, minorities, etc.)	Subtheme 6	0.773 (0.717 - 0.825)	0.513 (0.463 - 0.565)
12	Identify strategies that can be used to address the mental health burden of a pandemic in low mental health resource settings	Subtheme 6	0.771 (0.710 - 0.822)	0.525 (0.479 - 0.581)
13	Developing an accurate and reliable pandemic preparedness index to support LMIC in their pandemic preparedness planning	Subtheme 4	0.762 (0.700 - 0.815)	0.542 (0.489 - 0.588)
14	Identifying the challenges to supplying and delivering vaccines safely and efficiently in LMICs	Subtheme 6	0.760 (0.695 - 0.812)	0.550 (0.503 - 0.597)

Rank	Research idea	Subtheme	Overall RPS (95% CI)	Overall AEA-e (95% CI)
15	Designing behavioral strategies to improve public compliance and buy-in for infection control measures	Subtheme 3	0.760 (0.693 - 0.818)	0.577 (0.542 - 0.617)
16	Identifying effective strategies for high income countries (HICs) to expand their funding support to LMIC in improving pandemic preparedness	Subtheme 5	0.757 (0.700 - 0.810)	0.539 (0.490 - 0.584)
17	Evaluate approaches to improve technology capacity of vaccine development and equality of vaccine deployment in LMICs	Subtheme 6; Subtheme 7	0.756 (0.694 - 0.809)	0.548 (0.500 - 0.596)
18	Identifying the most effective data sharing and data use practices for epidemic surveillance for the emerging and re-emerging infections in LMIC	Subtheme 2	0.755 (0.691 - 0.809)	0.536 (0.483 - 0.583)
19	Developing a framework for mitigating a shortage of medical products/consumables and monitoring and notification of shortages	Subtheme 1	0.750 (0.684 - 0.806)	0.570 (0.538 - 0.616)
20	Assessing the effectiveness of Early Warning Outbreak Recognition System (EWORS) based on the One Health approach	Subtheme 2	0.747 (0.676 - 0.806)	0.538 (0.483 - 0.590)
21	Developing nutrition-sensitive social protection programs, particularly for disadvantaged groups (e.g., women and children, migrants), to prevent nutrition inequity during a pandemic	Subtheme 6	0.746 (0.686 - 0.802)	0.517 (0.474 - 0.565)
22	What is the relationship between the distribution of investments in health systems and the excess mortality observed during the pandemic?	Subtheme 4	0.746 (0.681 - 0.803)	0.515 (0.465 - 0.562)
23	Exploring how digital technologies can be used to improve healthcare access, aid healthcare workers, and improve information exchange	Subtheme 1; Subtheme 7	0.743 (0.680 - 0.797)	0.507 (0.459 - 0.557)
24	Identify approaches to modify the existing social support programs to lessen the burden of the pandemic on the poor and the vulnerable	Subtheme 6	0.742 (0.677 - 0.801)	0.539 (0.488 - 0.582)
25	Development and evaluation of effective interventions to increase pandemic literacy and engage communities in all settings	Subtheme 3	0.741 (0.676 - 0.796)	0.513 (0.465 - 0.559)
26	Developing and improving surveillance of the pathogens that cause pandemic, especially in LMIC	Subtheme 2	0.736 (0.674 - 0.794)	0.491 (0.443 - 0.536)
27	Developing innovative strategies to expand the existing health system capacities to accommodate patient influx for critical and acute care management	Subtheme 1	0.726 (0.664 - 0.784)	0.513 (0.463 - 0.553)
28	Evaluating the effectiveness of digital health in delivering health care services during strict restrictive measures	Subtheme 7	0.726 (0.662 - 0.786)	0.513 (0.468 - 0.551)
29	Conducting structured interviews with LMIC community members to identify most successful communication strategies during a pandemic	Subtheme 3	0.722 (0.659 - 0.780)	0.504 (0.459 - 0.545)
30	Identifying the areas of focus for the governments on strengthening their health systems for pandemic response	Subtheme 1	0.722 (0.658 - 0.780)	0.512 (0.465 - 0.550)

Rank	Research idea	Subtheme	Overall RPS (95% CI)	Overall AEA-e (95% CI)
31	Identifying social, economic, cultural, and political processes, including social drivers of vulnerability and risks, with the largest impacts on different communities in LMIC during a pandemic?	Subtheme 6	0.722 (0.657 - 0.781)	0.509 (0.462 - 0.556)
32	Study community empowerment as an effective approach to strengthen pandemic preparedness	Subtheme 4	0.722 (0.657 - 0.781)	0.500 (0.455 - 0.546)
33	Invest in studying the epidemiology of priority pathogens known to or having the potential for emerging as public health threats in LMIC	Subtheme 2	0.720 (0.654 - 0.780)	0.491 (0.444 - 0.538)
34	Assess the acceptability and feasibility of an innovative mobile-phone-based infectious disease surveillance system for healthcare workers in LMIC	Subtheme 7	0.718 (0.654 - 0.777)	0.507 (0.458 - 0.547)
35	Studying communication strategies and effective interventions to address misinformation and population's hesitancy towards evidence-based treatments	Subtheme 3	0.718 (0.653 - 0.780)	0.515 (0.469 - 0.553)
36	Identifying the essential elements required to form an effective pandemic response systems or teams	Subtheme 5	0.717 (0.653 - 0.778)	0.498 (0.455 - 0.546)
37	Developing criteria for identifying adequate preparedness of the country for the pandemic	Subtheme 4	0.716 (0.656 - 0.774)	0.465 (0.427 - 0.508)
38	Studying barriers to effective risk communication and health promotion strategies for key modifiable risks	Subtheme 3	0.716 (0.650 - 0.774)	0.516 (0.475 - 0.558)
39	Evaluating approaches to minimize vaccination and treatment hesitancy during a pandemic	Subtheme 3	0.714 (0.646 - 0.771)	0.524 (0.480 - 0.562)
40	Mapping the current state of pandemic preparedness in LMICs	Subtheme 4	0.714 (0.649 - 0.773)	0.473 (0.428 - 0.512)
41	Identify strategies and approaches for equitable health resources distribution during a pandemic	Subtheme 6	0.714 (0.650 - 0.773)	0.524 (0.479 - 0.559)
42	Studying ways to strengthen emergency alert and response teams with a particular focus on hard-to-reach areas	Subtheme 5	0.713 (0.650 - 0.772)	0.498 (0.454 - 0.539)
43	Studying the feasibility of developing low-cost data-driven disease surveillance systems in LMIC	Subtheme 2	0.712 (0.648 - 0.772)	0.472 (0.424 - 0.511)
44	Developing risk assessment tools suitable for adoption by most LMIC to prepare for the next pandemic	Subtheme 2	0.712 (0.644 - 0.771)	0.549 (0.523 - 0.584)
45	Evaluate approaches to promoting patient-centered integrated care to protect high-risk groups in times of pandemics	Subtheme 1	0.711 (0.642 - 0.767)	0.523 (0.481 - 0.561)
46	Designing methods to prioritize activities for pandemic preparedness in LMICs, given the limited resources that these countries have at their disposal	Subtheme 4	0.710 (0.644 - 0.770)	0.533 (0.489 - 0.568)

Rank	Research idea	Subtheme	Overall RPS (95% CI)	Overall AEA-e (95% CI)
47	Development of a health surveillance system with the use of GIS Mapping Software to identify an epidemic or pandemic as early as possible	Subtheme 2	0.710 (0.645 - 0.767)	0.447 (0.406 - 0.489)
48	Evaluating the experience of the past pandemic to improve allocation of health care resources to reduce avoidable deaths	Subtheme 2; Subtheme 4	0.708 (0.646 - 0.770)	0.512 (0.472 - 0.550)
49	Developing effective strategies for outreach workers - CHW to raise awareness on prevention (e.g., door to door vs. technology/based approach)	Subtheme 3	0.706 (0.642 - 0.765)	0.487 (0.442 - 0.530)
50	Studying the key success factors and barriers for development and implementation of countries' preparedness plans	Subtheme 4	0.705 (0.638 - 0.765)	0.502 (0.457 - 0.542)
51	Assessing the use of telemedicine in LMICs and the population groups falling into "the digital divide"	Subtheme 7	0.705 (0.639 - 0.763)	0.510 (0.459 - 0.549)
52	Identifying elements of national pandemic preparedness plans where LMIC countries need early and rapid international development support	Subtheme 4	0.703 (0.637 - 0.761)	0.509 (0.472 - 0.549)
53	Analyzing barriers to vaccine uptake by different social strata and in different settings and population groups, particularly in LMIC	Subtheme 3; Subtheme 6	0.703 (0.637 - 0.765)	0.510 (0.467 - 0.548)
54	Developing strategies to make any new vaccines affordable to all countries	Subtheme 6	0.703 (0.636 - 0.760)	0.487 (0.442 - 0.527)
55	Studying approaches to organize public health emergency response systems at the national level	Subtheme 4; Subtheme 5	0.701 (0.634 - 0.760)	0.499 (0.454 - 0.541)
56	Increasing media literacy among the general population to prevent the spread of conspiracy theories	Subtheme 3	0.699 (0.634 - 0.759)	0.455 (0.415 - 0.497)
57	Development of vaccines and therapeutics focusing on those diseases within WHO's list which have great epidemic potential	Subtheme 7	0.696 (0.631 - 0.753)	0.483 (0.439 - 0.523)
58	Studying the effectiveness of advocacy approaches to integrate pandemic preparedness in public health budgets at the global and national levels	Subtheme 4	0.695 (0.626 - 0.758)	0.496 (0.446 - 0.534)
59	Identify the effective pandemic/epidemic emergency preparedness training programs among healthcare workers in LMIC	Subtheme 1	0.694 (0.627 - 0.755)	0.506 (0.462 - 0.543)
60	Study the effectiveness of comprehensive national emergency communication plans, with identified channels, messages, bearers, to enable rapid deployment	Subtheme 3	0.693 (0.627 - 0.751)	0.505 (0.460 - 0.544)
61	What is the role of global governance in strengthening decision-making in low-and-middle-income countries (LMIC)?	Subtheme 4	0.693 (0.629 - 0.754)	0.464 (0.424 - 0.508)
62	Strengthening intervention strategy for supply chain through local production of drugs and vaccines in LMICS.	Subtheme 6	0.690 (0.623 - 0.749)	0.503 (0.457 - 0.539)
63	Identifying effective strategies to counter dis-information around pandemic response	Subtheme 3	0.689 (0.622 - 0.750)	0.512 (0.474 - 0.550)

Rank	Research idea	Subtheme	Overall RPS (95% CI)	Overall AEA-e (95% CI)
64	Assess innovative measures that could be taken to increase health workers' well-being, motivation and workplace safety during rapid surge of cases	Subtheme 1	0.688 (0.621 - 0.750)	0.495 (0.451 - 0.530)
65	Developing legal frameworks, regulatory instruments, and financing mechanisms to support outbreak prevention and deployment of countermeasures	Subtheme 4	0.687 (0.623 - 0.749)	0.488 (0.446 - 0.527)
66	Develop effective approaches to build trust across society and between countries to enable international scientific collaboration, evidence-based policy and adherence to recommendations	Subtheme 5	0.682 (0.617 - 0.744)	0.456 (0.420 - 0.496)
67	Evaluating effective approaches to ensure functional social services for the future pandemics	Subtheme 4	0.682 (0.618 - 0.742)	0.472 (0.431 - 0.511)
68	Evaluate approaches for establishing and strengthening treatment/care pathways for disease outbreaks in LMICs	Subtheme 1	0.681 (0.612 - 0.745)	0.500 (0.458 - 0.538)
69	Identification of factors that enhance poor disease notification and response and mitigation strategies in LMICS	Subtheme 2	0.681 (0.612 - 0.743)	0.508 (0.469 - 0.543)
70	Identify the most effective and equitable strategies for community-involvement in pandemic preparedness	Subtheme 6	0.678 (0.610 - 0.741)	0.509 (0.470 - 0.542)
71	Understanding factors affecting integration of community centered approaches to improve pandemic preparedness in low- and middle-income countries	Subtheme 5	0.675 (0.608 - 0.736)	0.476 (0.436 - 0.517)
72	Develop effective approaches for knowledge translation from academic institutions to policy in LMICs during a pandemic	Subtheme 4	0.675 (0.606 - 0.738)	0.496 (0.452 - 0.533)
73	Increasing knowledge and awareness of infectious diseases with pandemic potential among the general population	Subtheme 3	0.674 (0.610 - 0.735)	0.498 (0.457 - 0.535)
74	Identify strategies and measures to ensure that other health conditions are not neglected in LMICs due to a pandemic	Subtheme 1;Subtheme 6	0.672 (0.607 - 0.734)	0.506 (0.465 - 0.541)
75	Studying why were some LMIC countries seemingly relatively spared of COVID-19?	Subtheme 2	0.668 (0.602 - 0.731)	0.466 (0.425 - 0.504)
76	Identifying effective approaches for networking with neighboring countries and for global collaboration in pandemic response	Subtheme 5	0.667 (0.601 - 0.730)	0.473 (0.436 - 0.512)
77	Improving routine disease surveillance, identification and reporting system in both humans and animals for potential pathogens in both rural and urban health facilities	Subtheme 2	0.665 (0.601 - 0.725)	0.487 (0.449 - 0.522)
78	Studying cost-effective ways to strengthen primary healthcare centres with a particular focus on laboratory capacity	Subtheme 1	0.664 (0.597 - 0.725)	0.456 (0.420 - 0.491)
79	Exploring the use of mHealth to continue to provide key health services (e.g., ANC, immunisations, etc.) while limiting the risk of infection	Subtheme 7	0.662 (0.595 - 0.727)	0.493 (0.456 - 0.529)

Rank	Research idea	Subtheme	Overall RPS (95% CI)	Overall AEA-e (95% CI)
80	Identifying effective strategies to debunk fake news and misleading pandemic threat discourse in in low- and middle-income countries?	Subtheme 3	0.660 (0.590 - 0.722)	0.457 (0.421 - 0.493)
81	Identifying the population subgroups at high risk of pandemic spread in LMIC	Subtheme 2	0.658 (0.591 - 0.720)	0.512 (0.474 - 0.536)
82	Identifying the most effective strategies to prevent and control the emerging and re-emerging disease in LMIC in the abseence of vaccines and medicines	Subtheme 2	0.656 (0.590 - 0.719)	0.476 (0.442 - 0.513)
83	Studying how global financing should be strengthened for pandemic preparedness in low- and middle-income countries (LMICs).	Subtheme 4	0.655 (0.588 - 0.719)	0.482 (0.445 - 0.517)
84	Exploring approaches to quickly foster large global collaborations for pandemic prevention and control	Subtheme 5	0.655 (0.588 - 0.717)	0.477 (0.441 - 0.510)
85	Identifying the effective approaches to improve health system resilience to withstand pandemic shocks	Subtheme 1	0.653 (0.585 - 0.716)	0.468 (0.425 - 0.501)
86	Evaluate ways to achieve deep integration of information technology into the surveillance and management of pandemics in LMICs	Subtheme 7	0.652 (0.584 - 0.716)	0.450 (0.408 - 0.486)
87	Develop effective strategies to strengthen school health and nutrition programming to minimize disruption to children and adolescents in LMICs during a pandemic	Subtheme 1; Subtheme 6	0.652 (0.587 - 0.713)	0.470 (0.432 - 0.503)
88	Building capacity, demand and recommendations for telemedicine to assist healthcare provision during a pandemic	Subtheme 7	0.652 (0.585 - 0.719)	0.482 (0.442 - 0.514)
89	Reconfiguring the Global Financing Facility (GFF) to ensure health systems in LMICs become more resilient	Subtheme 4	0.651 (0.582 - 0.717)	0.427 (0.394 - 0.468)
90	Identifying the potential risk factors that can cause the emerging and re-emerging epidemic diseases in LMIC	Subtheme 2	0.649 (0.579 - 0.713)	0.508 (0.470 - 0.531)
91	Evaluating national-level approaches to improving public trust in science for the next pandemic	Subtheme 3	0.645 (0.581 - 0.708)	0.440 (0.408 - 0.474)
92	Studying how research and development (R&D) systems of LMICs can be developed to address potential future pandemics	Subtheme 7	0.641 (0.572 - 0.707)	0.485 (0.444 - 0.519)
93	Evaluating the development of an international database of strains for better cross-county collaboration for mitigation efforts	Subtheme 2; Subtheme 5	0.640 (0.574 - 0.709)	0.471 (0.433 - 0.504)
94	Studying risk behaviors and risk activities that should be restricted to prevent zoonotic spillover events in settings at the frontlines of disease emergence.	Subtheme 2	0.64 0(0.574 - 0.701)	0.442 (0.406 - 0.476)
95	Identifying the building blocks of the optimal information systems in different contexts to collect, process and present pandemic health data	Subtheme 7	0.640 (0.571 - 0.703)	0.464 (0.426 - 0.499)

Rank	Research idea	Subtheme	Overall RPS (95% CI)	Overall AEA-e (95% CI)
96	Developing strategies to incorporate indigenous knowledge systems around the world in preventing and dealing with new disease outbreaks	Subtheme 6	0.639 (0.571 - 0.702)	0.422 (0.389 - 0.456)
97	Identifying effective strategies for raising awareness in HICs that LMICs also need to be protected in the pandemic, or otherwise no-one is safe	Subtheme 3	0.639 (0.566 - 0.705)	0.449 (0.409 - 0.481)
98	Evaluate the most effective approaches for the global health actors to help LMICs to vaccinate their populations	Subtheme 5	0.636 (0.569 - 0.699)	0.479 (0.444 - 0.512)
99	How to rapidly evaluate cost-effectiveness of different diagnostic tests for massive screening during the pandemic?	Subtheme 2	0.635 (0.570 - 0.697)	0.495 (0.461 - 0.525)
100	Evaluate the use of the existing live vaccines (e.g., BCG, measles, oral polio, and possibly others) to enhance immune preparedness and reduce severity of a new pandemic	Subtheme 1	0.633 (0.560 - 0.698)	0.503 (0.464 - 0.526)
101	Strengthening psychological support for those in need through the development of more intelligent mobile solutions	Subtheme 7	0.632 (0.568 - 0.698)	0.446 (0.408 - 0.477)
102	Optimizing the implementation of advanced genome sequencing technologies for surveillance of emerging and re-emerging diseases	Subtheme 2	0.632 (0.558 - 0.702)	0.456 (0.415 - 0.495)
103	Discovery and development of new drugs to combat pathogens from natural and traditional medicines in LMICs	Subtheme 7	0.631 (0.564 - 0.692)	0.441 (0.405 - 0.473)
104	Studying how best to integrate and coordinate pandemic preparedness (including EWARS) and response strategies and policies within existing health systems structures	Subtheme 1; Subtheme 5	0.630 (0.560 - 0.698)	0.436 (0.401 - 0.470)
105	Identifying strategies to incentivize adoption of behavioral interventions (masking, social distancing) at population level for an extended period of time	Subtheme 3	0.629 (0.559 - 0.693)	0.476 (0.439 - 0.505)
106	Identifying the most efficient ways to translate knowledge into policy action in times of a pandemic	Subtheme 4	0.628 (0.560 - 0.690)	0.468 (0.430 - 0.500)
107	Forming an Advisory Group of experts with representatives from industry, academia, and international organisations that can be rapidly scaled in any pandemic	Subtheme 5	0.622 (0.554 - 0.684)	0.428 (0.394 - 0.461)
108	Studying how the health systems react to a crisis with limited resources and how they achieve antifragility	Subtheme 1	0.621 (0.554 - 0.688)	0.481 (0.442 - 0.509)
109	Identify approaches that can effectively prevent endemic diseases from becoming a pandemic	Subtheme 2	0.621 (0.551 - 0.687)	0.473 (0.435 - 0.501)
110	Understanding challenges and opportunities in funding coordination for public health emergencies in low-and middle-income countries	Subtheme 4	0.613 (0.545 - 0.678)	0.458 (0.422 - 0.491)
111	Developing rapid systems for testing the non-specific effects of future pandemic vaccines	Subtheme 2	0.609 (0.532 - 0.679)	0.458 (0.423 - 0.493)

Rank	Research idea	Subtheme	Overall RPS (95% CI)	Overall AEA-e (95% CI)
112	Identifying approaches and methods to increase modeling capacity and accuracy to predict impact of pandemic and inform policy	Subtheme 2	0.607 (0.536 - 0.678)	0.453 (0.414 - 0.484)
113	Identifying the level of awareness of health professionals at primary health care facilities with regard to pandemic preparedness	Subtheme 1	0.604 (0.537 - 0.672)	0.451 (0.419 - 0.484)
114	Establishing the effectiveness of the current communication system in LMIC in relaying real-time information between the decision makers and front-liners	Subtheme 2; Subtheme 3	0.602 (0.531 - 0.665)	0.422 (0.393 - 0.453)
115	Enhancing capacity to conduct multi-pathogen surveillance studies as part of pandemic preparedness	Subtheme 2	0.602 (0.530 - 0.668)	0.438 (0.403 - 0.469)
116	Increasing the number of safe houses for women and children to prevent them from being constrained at homes with violent people, alcoholics and/or drug abusers	Subtheme 1	0.602 (0.534 - 0.669)	0.476 (0.441 - 0.503)
117	Analysing if the WHO Checklist for pandemic preparedness is a valid and useful tool for LMIC	Subtheme 2	0.598 (0.529 - 0.661)	0.477 (0.440 - 0.503)
118	Development of reliable, rapid and low-cost tests for detection of multiple pathogens in wastewater	Subtheme 7	0.598 (0.529 - 0.668)	0.435 (0.401 - 0.466)
119	Which factors can enhance the global programme for combating pandemics in LMIC	Subtheme 5	0.597 (0.528 - 0.664)	0.448 (0.413 - 0.480)
120	Identifying best approaches to evaluate the use of new vaccines under emergency use authorization during a pandemic by weighing the risks and benefits	Subtheme 2	0.596 (0.526 - 0.659)	0.417 (0.384 - 0.448)
121	Can artificial intelligence in healthcare (i.e. machine learning algorithms) be utilized to predict patients outcome and facilitate management of healthcare resources?	Subtheme 7	0.595 (0.528 - 0.662)	0.418 (0.385 - 0.449)
122	Strengthening the care for the elderly by using personalized wearables, serving both to monitor health status and to communicate with family members	Subtheme 7	0.594 (0.527 - 0.658)	0.433 (0.400 - 0.464)
123	Evaluating the impact of the integrated disease surveillance and response (IDSR) framework (launched in 1988) on the pandemic preparedness in LMICs	Subtheme 2	0.593 (0.520 - 0.666)	0.463 (0.424 - 0.494)
124	Identify effective Interventions that promote early childhood development during the strict pandemic control measures, especially in LMIC	Subtheme 1	0.590 (0.521 - 0.660)	0.429 (0.395 - 0.459)
125	Developing guidelines to refer to outbreaks of diseases wiithout profiling any races. ethnicities, or geographic locations	Subtheme 3	0.588 (0.513 - 0.657)	0.458 (0.421 - 0.488)
126	Developing frameworks for sustainable health post-pandemic for a country to follow	Subtheme 4	0.587 (0.516 - 0.655)	0.438 (0.402 - 0.467)

Rank	Research idea	Subtheme	Overall RPS (95% CI)	Overall AEA-e (95% CI)
127	Educating people on solidarity and why the care for others in the community is important, especially those most vulnerable	Subtheme 3	0.587 (0.518 - 0.653)	0.441 (0.405 - 0.472)
128	Redesigning routine services to ensure that they remain unaffected in a future lockdown	Subtheme 1	0.585 (0.515 - 0.649)	0.493 (0.460 - 0.511)
129	Identify the challenges for communicating about a pandemic at its onset so that it facilitates better preparedness	Subtheme 3	0.579 (0.508 - 0.645)	0.481 (0.450 - 0.507)
130	How can antimicrobial stewardship programs be improved in LMIC?	Subtheme 1	0.574 (0.508 - 0.641)	0.417 (0.387 - 0.449)
131	Evaluating crowdsourcing and predictive machine learning analytics as a low-cost surveillance of infectious disease during a pandemic	Subtheme 7	0.574 (0.505 - 0.646)	0.441 (0.408 - 0.470)
132	Model the potential effect of reducing the digital divide on tackling social and health inequalities intensified by the pandemic	Subtheme 6; Subtheme 7	0.573 (0.506 - 0.643)	0.434 (0.397 - 0.463)
133	Identify strategies for maintaining the balance between epidemiological effectiveness and financial optimization of a healthcare system in a LMIC country	Subtheme 4	0.572 (0.497 - 0.641)	0.441 (0.405 - 0.470)
134	Using mixed methods to improve capacity and infrastructure for pandemic preparedness in LMICs	Subtheme 1	0.572 (0.503 - 0.641)	0.472 (0.437 - 0.497)
135	Studying the willingness of national health authorities in LMIC to comply with the WHO's recommendations on epidemics surveillance	Subtheme 2; Subtheme 4	0.569 (0.495 - 0.632)	0.428 (0.398 - 0.458)
136	Expanding efforts to identifying pathogens that can cause pandemic at the global level	Subtheme 2	0.567 (0.498 - 0.631)	0.420 (0.389 - 0.448)
137	Exploring the association between climate, seasonability and viral transmissibility	Subtheme 2	0.565 (0.495 - 0.634)	0.436 (0.404 - 0.464)
138	Evaluating strategies to prevent economic collapse while dealing with a new pandemic	Subtheme 4	0.563 (0.492 - 0.631)	0.442 (0.414 - 0.473)
139	Exploring how can environment facilitate rapid spread of a pathogen	Subtheme 2	0.563 (0.495 - 0.628)	0.438 (0.407 - 0.468)
140	Develop targeted and context-specific measures to support micro, small and medium-sized agri-businesses to ensure food system inclusiveness, adaptation, and resilience in LMICs	Subtheme 6	0.561 (0.489 - 0.627)	0.435 (0.404 - 0.469)
141	Identify the challenges for effective transportation of patients to health care and establishing a local ambulance service in LMICs	Subtheme 1	0.559 (0.487 - 0.625)	0.461 (0.426 - 0.490)
142	What should be the personal and professional characteristics of health system leaders who are at the forefront of decision-making in the face of pandemics?	Subtheme 1; Subtheme 4	0.553 (0.485 - 0.622)	0.437 (0.406 - 0.463)
143	Studying the governance mechanisms for development and implementation of preparedness plans in LMIC	Subtheme 4	0.551 (0.482 - 0.617)	0.431 (0.399 - 0.459)

Rank	Research idea	Subtheme	Overall RPS (95% CI)	Overall AEA-e (95% CI)
144	Identify the scope for behavioral science to address sustainable health post-pandemic	Subtheme 3	0.548 (0.476 - 0.618)	0.460 (0.427 - 0.487)
145	Studying cost-effectiveness of the WHO's presented recommendations on epidemiological surveillance in LMICs	Subtheme 2	0.539 (0.466 - 0.609)	0.460 (0.426 - 0.486)
146	Identify the lessons learnt from any previous population screening so that it helps medical services, logistics and other non-health counterparts	Subtheme 2	0.529 (0.458 - 0.597)	0.452 (0.422 - 0.483)
147	Creating online educational content for pupils of all ages in advance, so that the school can be held remotely in case of need	Subtheme 7	0.525 (0.456 - 0.589)	0.468 (0.431 - 0.488)
148	Training, enlisting and legally enabling alternative and complementary medicine practitioners to respond to pandemic emergencies	Subtheme 1	0.524 (0.453 - 0.595)	0.467 (0.435 - 0.492)
149	Studying if people abandon protective measures too early after vaccination and how do their behavioral responses affect the outcome of government's mitigation efforts	Subtheme 2; Subtheme 3	0.521 (0.450 - 0.586)	0.428 (0.398 - 0.458)
150	Analysis and mapping resilience of health care systems and all of their key components	Subtheme 1	0.515 (0.445 - 0.587)	0.446 (0.413 - 0.476)
151	Identifying roles and responsibilities of high(er) income countries in improving pandemic preparedness for LMICs	Subtheme 5	0.515 (0.448 - 0.580)	0.435 (0.401 - 0.461)
152	Develop strategies to sensitize policy-makers at government level to the usefulness of wastewater-based epidemiology as a complementary tool to epidemiological surveillance	Subtheme 4	0.512 (0.439 - 0.584)	0.428 (0.390 - 0.455)
153	Identify the most effective strategies for moving climate change and disaster preparedness to the top of political agenda in all countries	Subtheme 4	0.511 (0.444 - 0.583)	0.475 (0.438 - 0.500)
154	Evaluate the effective approaches to address stigma relating to mental illness in LMICs during a pandemic	Subtheme 3	0.507 (0.443 - 0.577)	0.448 (0.414 - 0.472)
155	Identifying best approaches for strengthening primary health care through the introduction of "chat bots"	Subtheme 7	0.500 (0.429 - 0.574)	0.448 (0.410 - 0.474)
156	Developing international guidelines and algorithms for online work methods for schooling and for training of teachers	Subtheme 7	0.497 (0.431 - 0.564)	0.414 (0.382 - 0.439)
157	Assess the potential long-term adverse health events associated with vaccines to reassure the public and reduce vaccine hesitancy	Subtheme 2; Subtheme 3	0.485 (0.414 - 0.553)	0.460 (0.429 - 0.486)
158	Studying how to raise the public's awareness of the importance of strengthening the capacity of global research to prevent future pandemics	Subtheme 3	0.485 (0.418 - 0.552)	0.441 (0.412 - 0.469)
159	Using mixed methods to better understand global health threat with pandemic potential, such as COVID 19	Subtheme 2	0.484 (0.416 - 0.555)	0.444 (0.410 - 0.467)
160	Identifying strategies to improve strategic knowledge generation and decision-making dissemination for healthcare and research in LMICs	Subtheme 4	0.456 (0.387 - 0.526)	0.453 (0.418 - 0.481)

Rank	Research idea	Subtheme	Overall RPS (95% CI)	Overall AEA-e (95% CI)
161	Investigating career satisfaction among frontline healthcare practitioners during a pandemic	Subtheme 1	0.342 (0.280 - 0.408)	0.439 (0.401 - 0.473)
162	Exploring if sustainable development goals in LMICs are still attainable by 2030?	Subtheme 4	0.332 (0.271 - 0.397)	0.444 (0.408 - 0.481)
163	Defining an epidemic wave in a standardized manner during a pandemic	Subtheme 2	0.317 (0.255 - 0.387)	0.485 (0.440 - 0.524)

Note: 95% CI, 95% confidence interval; RPS, Research Priority Scores; AEA-e, average expert agreement score based entropy; Subtheme 1: improving health system capacity and resilience; Subtheme 2: enhancing surveillance, monitoring and evaluation; Subtheme 3: improving risk communication and health promotion; Subtheme 4: fostering policy, planning and decision making; Subtheme 5: improving coordination and collaboration; Subtheme 6: promoting equitable access; Subtheme 7: fostering innovations and new technologies.

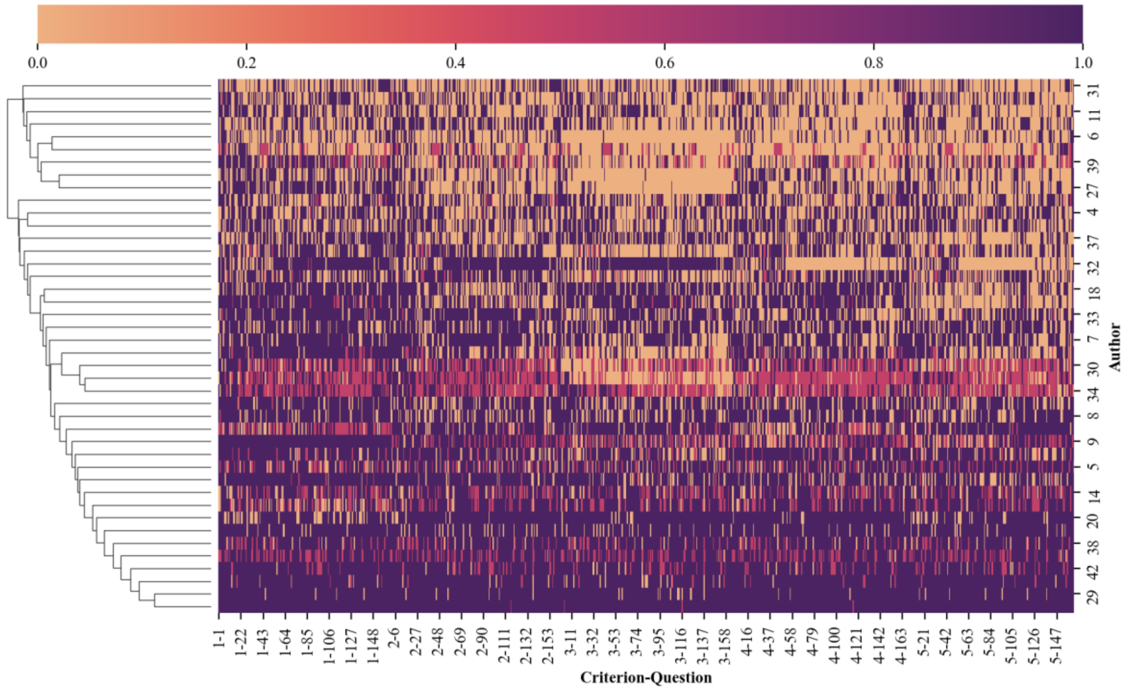

Figure S1. The hierarchical clustering of all idea scores from contributing experts

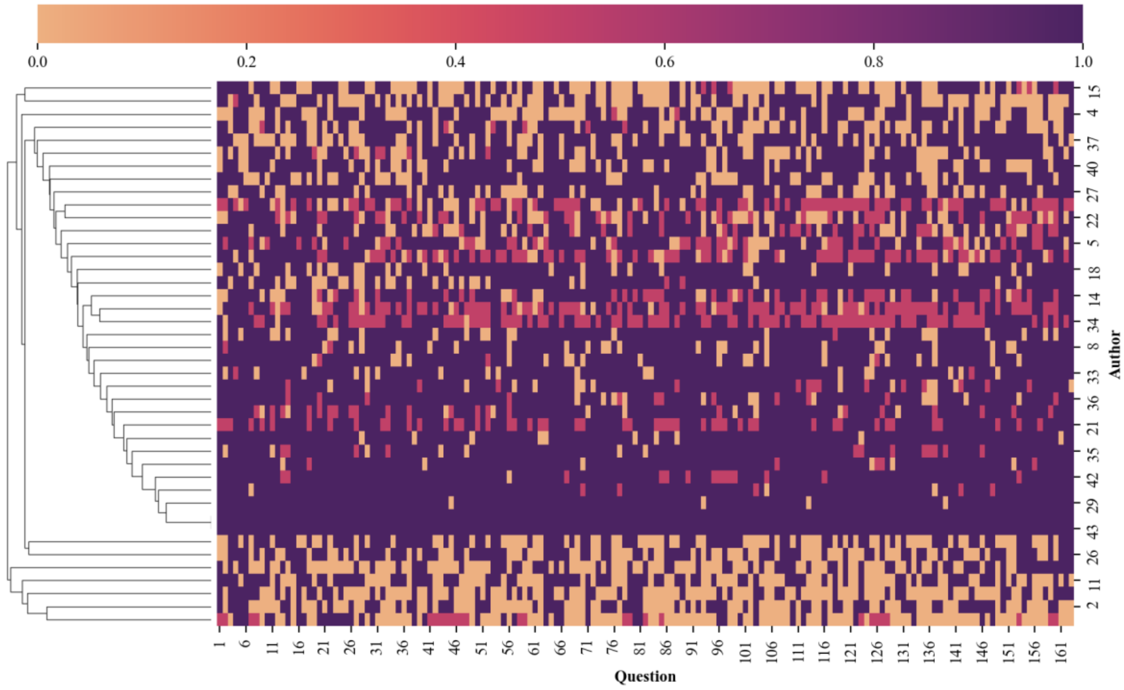

Figure S2. The clustering of idea scores in the criterion-"feasibility and answerability"

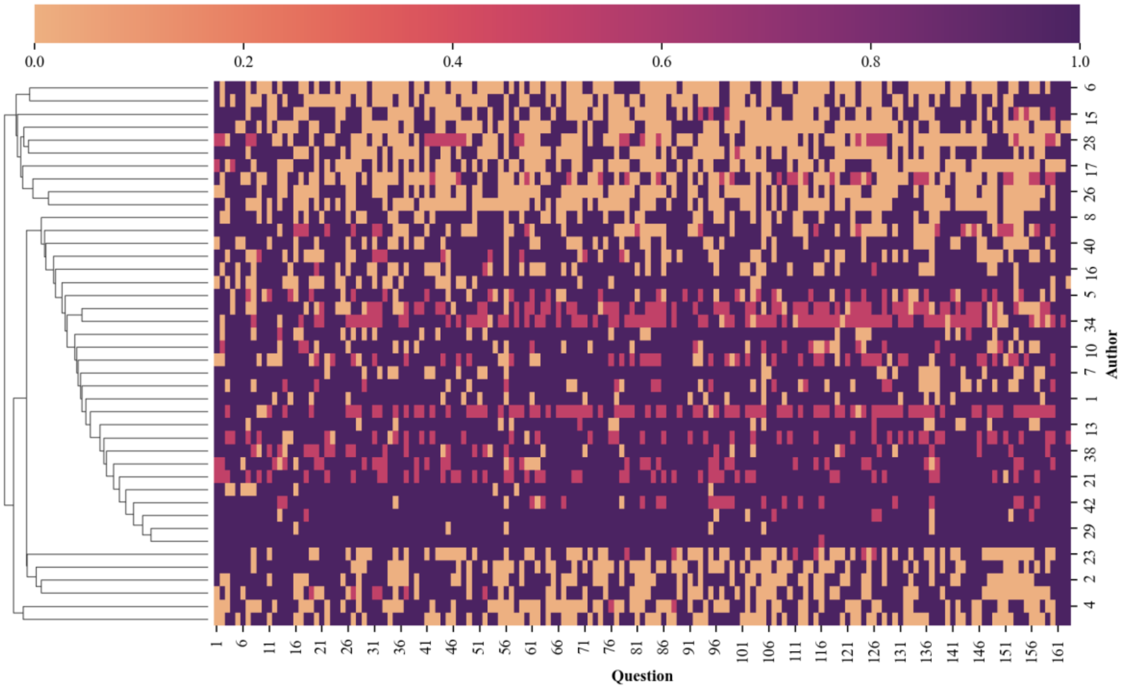

Figure S3. The clustering of idea scores in the criterion-"impact on burden reduction　　"

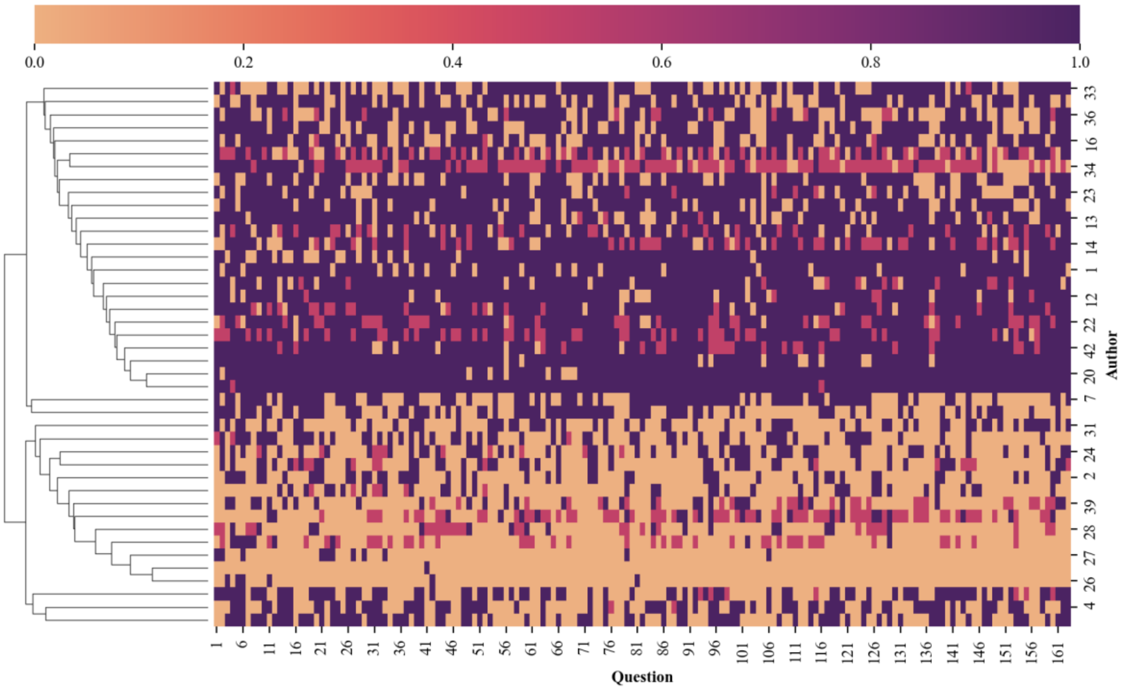

Figure S4. The clustering of idea scores in the criterion-"potential for paradigm shift"

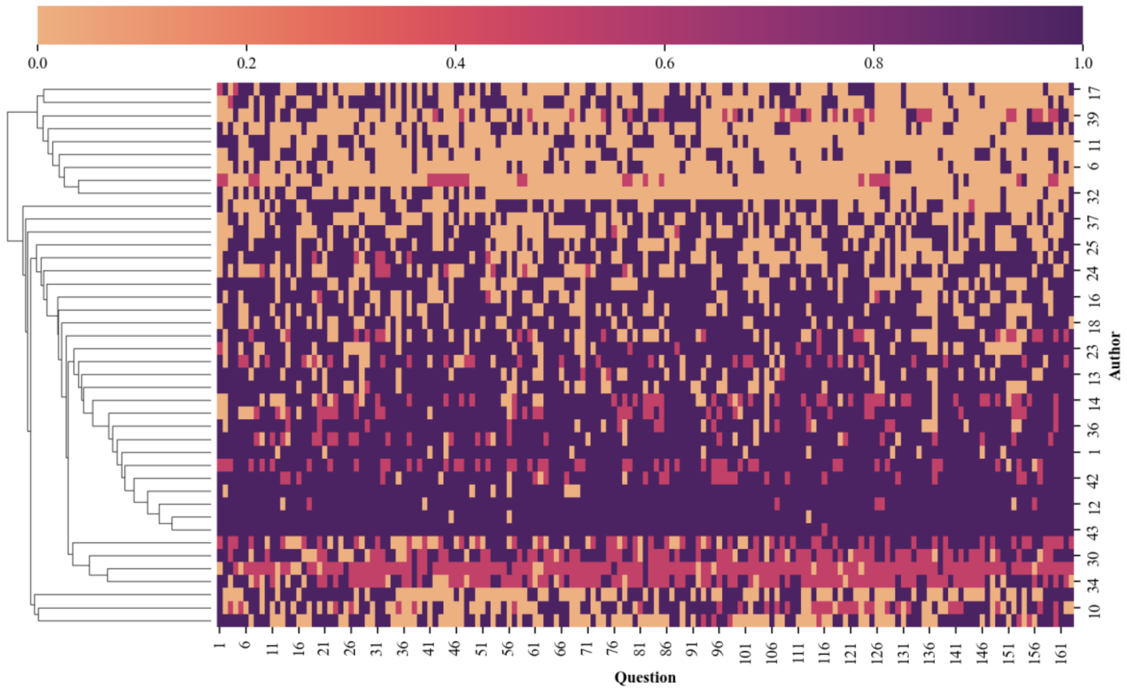

Figure S5. The clustering of idea scores in the criterion-"potential for translation and implementation"

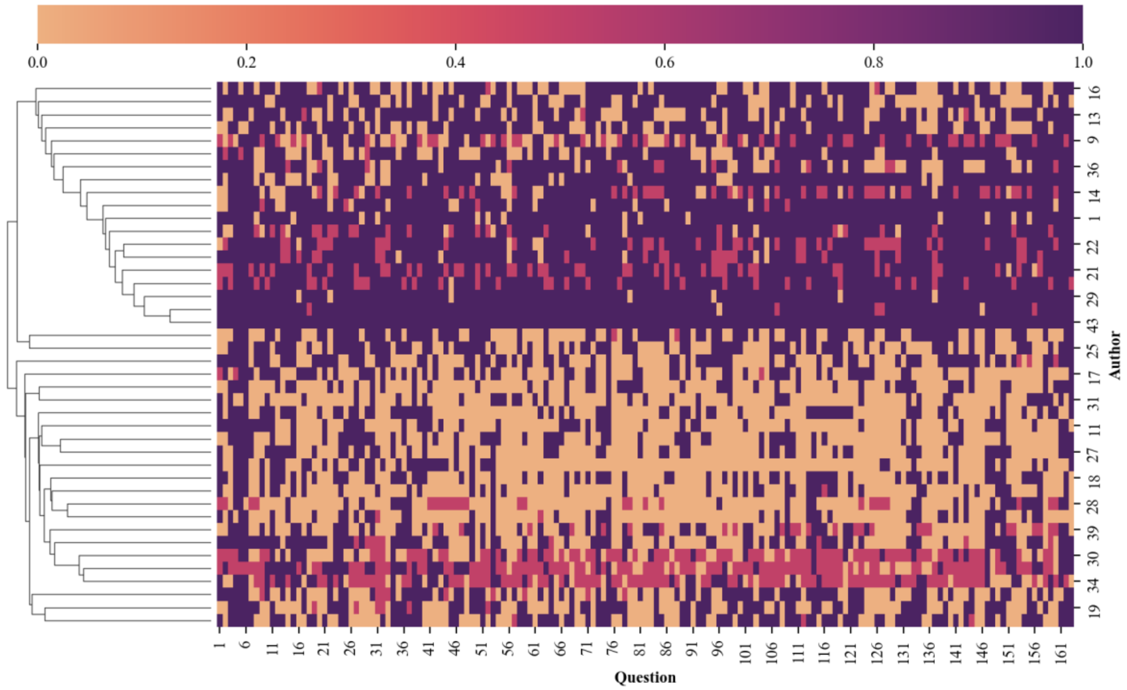

Figure S6. The clustering of idea scores in the criterion-"impact on equity"
